# Pimarane Diterpenoids from Aerial Parts of *Lycopus lucidus* and Their Antimicrobial Activity

**DOI:** 10.1155/2022/5178880

**Published:** 2022-02-04

**Authors:** Jitendra Pandey, Bang Yeon Hwang, Hyeong-Kyu Lee, Amrit Poudel

**Affiliations:** ^1^Department of Pharmacy, Crimson College of Technology, Pokhara University, Devinagar-11, Butwal 32900, Nepal; ^2^Chungbuk National University, Department of Pharmacy, Cheongju, Republic of Korea; ^3^Natural Medicine Research Center, Korea Research Institute of Bioscience & Biotechnology, Cheongju-si 363-883, Republic of Korea; ^4^Department of Biodiversity and Bioresources, Satvik Nepal, Dandakonak, Kaski, Pokhara 33700, Nepal

## Abstract

The ethyl acetate fraction obtained from aerial parts of *L. lucidus* was subjected for isolation of new bioactive compounds, which enabled isolation of five new pimarane-type diterpenoids, namely, 3*β,* 8*β,* 12*β*, 18-tetrahydroxy pimar-15-ene **(10),** 7*α,* 8*β,* 12*β*, 18-tetrahydroxy pimar-15-ene **(11),** 3*β,* 8*β,* 11*β,* 12*α*, 18-pentahydroxy pimar-15-ene **(12),** 12*β* acetoxy, 8*β,* 3*β*, 18-trihydroxy pimar-15-ene **(13),** and 3*β* acetoxy, 8*β,* 12*β,* 18-trihydroxy pimar-15-ene **(14),** along with nine known compounds. The structures were elucidated by spectroscopic analysis and comparison with literature data. The isolated new pimarane diterpenoids were examined for antimicrobial activity against Gram-negative and Gram-positive bacteria strains. Among them, the compound 3*β,* 8*β,* 12*β*, 18-tetrahydroxy pimar-15-ene **(10)** was most effective, exhibiting minimum inhibitory concentration (MIC) values of 15.62 µg/mL against *Staphylococcus epidermidis*, 31.25 *µ*g/mL against *Staphylococcus aureus*, 62.5 *µ*g/mL against *Pseudomonas aeruginosa*, and 125 *µ*g/mL against *Escherichia coli*.

## 1. Introduction

The genus *Lycopus* of family Lamiaceae (Labiatae) contains around 16 species with wide distribution in Europe, Asia, and North America [1]. In Asia, *Lycopus lucidus* is widely distributed species in Korea, China, Japan, Russia, and Taiwan. It is most abundant in Korea. *L. lucidus* is a flowering perennial glabrous herb occurring in aquatic environment and grows up to 0.6–1.2 m height at an altitude of 320 m to 2100 m [2–4]. *L. lucidus* is one of the popular edible plants with its long history as a folk remedy in traditional medicinal system of China, Japan, and Korea [5]. This plant has been used as both traditional and official formulations, as they are potent source of bioactive tannins, coumarins, flavonoids, terpenoids, and essential oils. Major bioactive compounds that have been isolated from this plant are flavonoid and its esters, rosmarinic acid derivatives, phenylpropanoids, steroids, pentacyclic triterpenes, essential oils, oligosaccharides, polysaccharides, and diterpenoid glycosides [3, 6–8]. The leaf and stem (aerial part) of *L. lucidus* have been extensively used for the treatment of inflammation, cardiovascular problem, insomnia, menstrual problems, and thyroid problem, as a sedative, wound healing, pain reliving agents, herbal tea, and useful tonic [6, 8, 9]. The root of *L. lucidus* is known as small ginseng in China and widely used as dietary supplement [10]. Many biological activities such as inhibition of superoxide radical [4], nitric oxide scavenging effect [6], inhibition of hypercholesterolemia and atherosclerosis [11], acaricidal activities [10], and hyaluronidase inhibition [5] have been explored from this plant.

In the current scenario, bacterial infectious diseases are a serious worldwide public health problem due to an increase in their resistance towards antibiotics, which have ultimately given on to the birth of multiresistant bacterial strains. Increased rates of mortality and morbidity are due to the lack of long-term effective drugs and the unaffordable cost of new generation antibiotics. The problem of microbial resistance is growing and the prospect of the use of antimicrobial drugs is uncertain. This disastrous situation has compelled us to explore more successful antimicrobial agents using plant resources so that they will serve as an active therapeutic ingredient, as well as leading molecule for the synthesis of optimized new drugs. The plant species have always been serving as a major source of novel and potent antimicrobial constituents, as they possess the capability to synthesize secondary metabolites to combat diverse pathogenic microorganisms available in the environment [12]. In this context, our study is mainly focused on isolation and structure elucidation of possible new compounds from the aerial parts of *L. lucidus* followed by screening their antibacterial properties against pathogenic Gram-positive and Gram-negative bacterial strains.

## 2. Materials and Methods

### 2.1. General Experimental Procedure

Analysis of NMR spectra was carried out through Varian UNITY 400 (Varian, Inc., Palo Alto, CA) FT-NMR spectrometer using the tetramethylsilane as an internal standard. Waters Q-Tof Premier spectrometer (Micromass UK Ltd., Manchester, UK) was used to obtain HR-ESI mass spectra. Sephadex LH-20 (25–100 *µ*m, Sigma-Aldrich, Steinheim, Germany), silica gel (230–400 mesh, SiliCycle Inc., Quebec, Canada), and RP-C18 (Cosmosil 40C_18_-PREP, Kyoto, Japan) were used for column chromatography. Thin layer chromatography (TLC) was performed on precoated Kiesel-gel 60 F_254_ (0.25 mm, Merck, Darmstadt, Germany) and Kiesel-gel 60 RP-18F_254s_ (0.25 mm, Merck, Steinheim, Germany). HPLC analysis was conducted on Thermo Dionex Ultimate 3000 equipped with Ultimate 3000 pump, autosampler, column compartment and diode array detector and Agilent Technology model 1260 Infinity equipped with 1260 DAD. Columns used for the analysis were YMC Trait C18 ExRS (5 *µ*m, 4.6 × 250 mm), Phenomenex synergy RP-Polar (5 *µ*m, 4.6 × 250 mm), Thermo acclaim polar advantage (5 *µ*m, 4.6 × 250 mm), and Atlantis T3 (5 *µ*m, 4.6 × 250 mm). Preparative HPLC was carried out with a model Gilson PLC 2020. Preparative columns were Thermo acclaim polar advantage (5 *µ*m, 21.2 × 250 mm), Atlantis (5 *µ*m, 19 × 250 mm), and YMC Trait C18 ExRS (5 *µ*m, 20 × 250 mm).

### 2.2. Plant Material

The aerial parts of *L*. *lucidus* were collected from ‘a private herb garden' in Yuseong-gu, Daejeon, Republic of Korea (geographical coordinates: 36.3622° N, 127.3561° E), in May 2016. The taxonomical identification of the plant was confirmed by Prof. Ki Hwan Bae, Chungnam National University. A voucher specimen (KRIB0071766) was deposited at the herbarium of the Korea Research Institute of Bioscience and Biotechnology (KRIBB).

### 2.3. Extraction and Isolation from Aerial Parts of L. lucidus

Air-dried aerial parts of *L*. *lucidus* (3.5 kg) were extracted five times by cold maceration with methanol (9 L) at room temperature, and 388 g of a solid extract was obtained. The methanol extract (388 g) was suspended in distilled water and then partitioned with hexane, ethyl acetate, and water-saturated butanol, which yielded 60 g of hexane-soluble extract, 49 g of ethyl acetate soluble extract, 45 g of butanol soluble extract, and 142 g of an aqueous extract. From the data of HPLC and TLC, the ethyl acetate fraction was shown to contain major compounds such as terpenoids and phenolics. The ethyl acetate fraction (35 g) was applied to reverse phase open column (10.5 cm × 35 cm, 2.8 kg of RP-C18 gel) and eluted with a stepwise gradient of methanol in distilled water (20%, 30%, 40%, 50%, 60%, 70% 80%, and 90% methanol, each 8 L), yielding 10 subfractions on the basis of TLC pattern (denoted by SR-EA-F1 to SR-EA-F10. Compound (**1)** (29 mg) was obtained from fraction SR-EA-F3 (455 mg) using Preparative Liquid Chromatography PLC (5%–50% acetonitrile, gradient, Thermo acclaim polar advantage 5 *µ*m, 250 × 21.2 mm). Compound (**2)** (13 mg) and compound (**3)** (11 mg) were separated from fraction SR-EA-F4 (579 mg) through PLC (5%-95 acetonitrile, gradient, Thermo acclaim polar advantage 5 *µ*M, 250 × 21.2 mm). The compound **(4)** (17 mg) was isolated from the fraction SR-EA-F5 (345 mg) using PLC again (7%-75 acetonitrile, gradient, Thermo acclaim polar advantage 5 *µ*M, 250 × 21.2 mm). Fraction SR-EA-F6 (412 mg) was subjected to Sephadex LH-20 (100% methanol as an eluent) to yield compound **(5)** (41 mg). PLC analysis (7%–75% acetonitrile gradient, YMC Trait C18 ExRS 5 *µ*M, 250 × 20 mm.) of fraction SR-EA-F7 (932 mg) resulted in impure fraction SR-EA-F7.1(575 mg) and pure compound (**8)** (15 mg). Fraction SR-EA-F7.1 again was subjected to PLC (5%–70% acetonitrile gradient, YMC Trait C18 ExRS 5 *µ*M, 250 × 20 mm) to give compound (**6)** (93 mg) and compound **(7)** (115 mg). Fraction SR-EA-F8 (899 mg) was subjected to Sephadex LH-20 (100% methanol as an eluent) to give UV active subfraction SR-EA-F8.1 (456 mg) and UV-nonactive subfraction SR-EA-F8.2 (220 mg). Compound (**9)** (123 mg) was obtained from subfraction SR-EA-F8.1 by PLC analysis (5%–95% acetonitrile gradient, YMC Trait C18 ExRS 5 *µ*M, 250 × 20 mm). From subfraction SR-EA-F8.2, compound (**10)** (125 mg) and compound (**11)** (2.5 mg) were obtained through normal phase open column chromatography (5 cm × 45 cm; 300 g of silica gel; hexane: EtOAc at 3 : 7) and fraction SR-EA-F9 (279 mg) and fraction SR-EA-F10 (310 mg) were also subjected to normal phase open column chromatography (5 cm × 45 cm; 300 g of silica gel; hexane: EtOAc at 4 : 6 and 5 cm × 45 cm; 300 g of silica gel; hexane: EtOAc at 1 : 1, respectively). Fraction SR-EA-F9 resulted in compound (**12)** (21 mg) whereas compound (**13)** (25 mg) and compound (**14)** (3 mg) were isolated from fraction SR-EA-F10 using same method used for compound (**13**).

#### 2.3.1. Protocatechuic Acid (1)

White gray crystalline powder; HR-ESI-MS *m/z*: 153.12[M-H]-. ^1^H-NMR (400 MHz, DMSO-*d*_6_) *δ*: 7.33 (1H, *d*, *J* *=* 2.0 Hz, H-2), 7.28 (1H, dd, *J* = 2.0, 8.2 Hz, H-6), 6.77 (H, *d*, *J* *=* 8.0 Hz, H-5). ^1^³C-NMR (100 MHz, DMSO- *d*_6_) *δ*: 167.3 (-COOH, C-7), 155.0 (C-4), 144.9 (C-3), 121.9 (C-5), 121.7 (C-1), 116.5 (C2), 115.1 (6).

#### 2.3.2. Protocatechualdehyde (2)

White gray crystalline powder; HR-ESI-MS *m/z*: 137.0251[M-H]-. ^1^H-NMR (400 MHz, DMSO-*d*_6_) *δ*: 9.69 (1H, s, H-7), 7.25 (1H, *d*, *J* = 8.0 Hz, H-6), 7.22 (1H, s, H-2), 6.90 (H, s, H-5). ^1^³C-NMR (100 MHz, DMSO- *d*_6_) *δ*: 190.0 (-CHO, C-7), 152.1 (C-4), 145.8 (C-3), 129.8 (C-5), 124.49 (C-1), 115.5 (C2), 114.3 (6).

#### 2.3.3. Methyl 3,4 Dihydroxy Benzoate (3)

White gray crystalline powder; HR-ESI-MS *m/z*: 167.148[M-H]-. ^1^H-NMR (400 MHz, DMSO-*d*_6_) *δ*: 7.43 (1H, dd, *J* *=* 2.0, 8.0z, H-6), 7.42 (1H, s, H-2), 6.83 (1H, *d*, *J* = 8.8 Hz, H-5), 3.801 (3H, s-OCH_3_, H-8). ^1^³C-NMR (100 MHz, DMSO-*d6*) *δ*: 167.12 (Carbonyl carbon, C-7), 151.01 (C-4), 147.18 (C-3), 123.44 (C-1), 121.74 (C-6), 114.98 (C-5), 112.71 (C-2), 55.526 (-OCH_3_, C-8).

#### 2.3.4. Para-Hydroxy Benzoic Acid (4)

White crystalline powder; HR-ESI-MS *m/z*: 137.12[M-H]-. ^1^H-NMR (400 MHz, DMSO-*d*_6_) *δ*: 7.78 (2H, *d*, *J* = 8.5 Hz, H-3& H-5), 6.81 (2H, *d*, *J* = 8.4 Hz, H-2& H-6). ^1^³C-NMR (100 MHz, DMSO-*d6*) *δ*: 167.1 (-COOH), 161.5 (C-4), 131.5 (C-2& C6), 121.4 (C-1), 115.0 (C-3&C5).

#### 2.3.5. Caffeic Acid (5)

White crystalline powder; HR-ESI-MS *m/z*: 179.159[M-H]-. ^1^H-NMR (400 MHz, Methanol-*d*_4_) *δ*: 7.55 (1H, *d*, *J* = 15.6 Hz, H-7), 7.07 (1H, *d*, *J* = 2.0 Hz, H-2), 6.95 (1H, dd, *J* = 2.0 Hz, 8.4 Hz H-6), 6.81 (1H, *d*, *J* = 8.4 Hz, H-5), 6.224 (1H, *d*, *J* = 15.6 Hz, H-8). ^1^³C-NMR (100 MHz, Methanol-*d*_4_) *δ*: 171.2 (C-9), 149.5 (C-4), 147.0 (C-7), 146.9 (C-3), 127.9 (C-1), 122.9 (C-6), 116.6 (C-5), 115.7 (C-8), 115.2 (C-2).

#### 2.3.6. Rosmarinic Acid (6)

White crystalline powder; HR-ESI-MS *m/z*: 359.29[M-H]-. ^1^H-NMR (400 MHz, Methanol-*d*_4_) *δ*: 7.51 (1H, *d*, *J* = 15.5 Hz, H-7′), 7.03 (1H, *d*, *J* = 2.0 Hz, H-2′), 6.82 (1H, dd, *J* = 2.0, 8.5 Hz, H-6′), 6.72 (1H, *d*, *J* *=* 8.5 Hz, H-5′), 6.66 (1H, *d*, *J* = 2.0 Hz, H-2), 6.64 (1H, *d*, *J* = 8.0 Hz, H-5), 6.57 (1H, dd, *J* = 2.0, 8 Hz, H-6), 6.28 (1H, *d*, *J* = 15.5 Hz, H-8′), 5.20 (1H, dd, *J* *=* 5.0, 7.5 Hz, H-8), 3.06 (1H, dd, *J* *=* 5.5, 14.5 Hz, H-7a), 3.00 (1H, dd, *J* = 5.5, 14.5 Hz, H-7b). ^1^³C-NMR (100 MHz, Methanol-*d*_4_) *δ*: 171.3 (C-9), 166.8 (C-9′), 149.1 (C-4′), 146..6 (C-7′), 146.4 (C-3′), 145.8 (C-3), 144.9 (C-4), 129.1 (C-1), 127.3 (C-1′), 121.5 (C-6′), 117.3 (C-6), 116.6 (C-2), 115.6 (C-5′), 116.4 (C-5), 114.3 (C-2′), 114.2 (C-8′), 73.8 (C-8), 37.0 (C-7).

#### 2.3.7. Methyl Rosmarinate (7)

White crystalline powder; HR-ESI-MS *m/z*: 373.24[M-H]-. ^1^H-NMR (400 MHz, Methanol-*d*_4_) *δ*: 7.55 (1H, *d*, *J* = 15.5 Hz H-7′), 7.04 (1H, *d*, *J* = 2 Hz, H-2′), 6.95 (1H, dd, *J* = 2.0, 8.5 Hz, H-6′), 6.78 (1H, *d*, *J* = 8.5 Hz, H-5′), 6.70 (1H, *d*, *J* = 2.0 Hz, H-2), 6.69 (1H,d, *J* = 8.0 Hz, H-5), 6.57 (1H, dd, *J* = 2.0, 8.0 Hz, H-6), 6.26 (1H, *d*, *J* = 15.5 Hz, H-8′), 5.19 (1H, dd, *J* = 5.0, 7.5 Hz, H-8), 3.7 (3H, s, -OCH_3_), 3.06 (1H, dd, *J* = 5.5, 14.5 Hz, H-7a), 3.00 (1H, dd, *J* = 5.5, 14.5 Hz, H-7b). ^1^³C-NMR (100 MHz, Methanol-*d*_4_) *δ*: 172.3 (C-9), 168.4 (C-9′), 149.9 (C-4′), 148.1 (C-7′), 146.9 (C-3′), 146.3 (C-3), 145.5 (C-4), 128.8 (C-1), 127.7 (C-1′), 123.3 (C-6′), 121.9 (C-6), 117.6 (C-2), 116.6 (C-5′), 116.4 (C-5), 115.3 (C-2′), 114.2 (C-8′), 74.8 (C-8), 52.8 (-OCH_3_), 38.0 (C-7).

#### 2.3.8. Quercetin 3-O-*β*-D-Glucopyranoside (8)

Yellow crystalline powder; HR-ESI-MS *m/z*: 463.371[M-H]-. ^1^H-NMR (400 MHz, Methanol-*d*_4_) *δ*: 7.71 (1H, *d*, *J* = 1.6 Hz, H-2′), 7.58 (1H, dd, *J* = 1.2, 8.4 Hz, H-6′), 6.87 (1H, *d*, *J* = 8.4 Hz, H-5′), 6.39 (1H, s, H-8), 6.20 (1H, s, H-6), 5.25 (1H,d, *J* = 7.6 Hz, galactose H-1), 3.37–3.60 (4H,m, sugar protons). ^1^³C-NMR (100 MHz, Methanol-*d*_4_) *δ*: 179.6 (C-4), 166.1 (C-7), 163.2 (C-5), 159.1 (C-2), 158.5 (C-9), 149.9 (C-4′), 146.0 (C-3′), 135.7 (C-3), 123.3 (C-6′), 123.2 (C-1′), 117.6 (C-5′), 116.1 (C-2′), 105.8 (C-10), 104.4 (galactose-C-1), 100.0 (C-6), 94.8 (C-8), 78.5 (galactose-C-3), 78.2 (galactose-C-5), 75.8 (galactose-C-2), 71.3 (galactose–C-4), 62.7 (galactose-C-6).

#### 2.3.9. Luteolin (9)

Yellow amorphous powder; HR-ESI-MS *m/z*: 185.24[M-H]-. ^1^H-NMR (400 MHz, DMSO- *d*_6_) *δ*: 12.97 (s,-OH), 7.42 (1H, *d*, *J* = 1.6 Hz, H-6′), 7.39 (1H, s, H-2′), 6.88 (1H, *d*, *J* = 8.4 Hz, H-5′), 6.66 (1H, s, H-3), 6.43 (1H, s, H-8), 6.18 (1H, s, H-6). ^1^³C-NMR (100 MHz, DMSO- *d*_6_) *δ*: 181.6 (C-4), 164.1 (C-7), 163.8 (C-2), 161.4 (C-5), 157.2 (C-9), 149.7 (C-4′), 145.7 (C-3′), 121.4 (C-1′), 118.9 (C-6′), 115.9 (C-5′), 113.3 (C-2′), 103.6 (C-10), 102.8 (C-3), 98.82 (C-6), 93.8 (C-8).

#### 2.3.10. 3*β*, 8*β*, 12*β*, 18-Tetrahydroxy Pimar-15-en (10)

White crystalline needles; freely soluble in methanol and ethyl acetate; HR-ESI-MS [M-H]- ion at *m/z* 337.2354 and molecular formula C_20_H_34_O_4._^13^C (100 MHz, MeOD) and ^1^H-NMR (400 MHz, MeOD) spectroscopic data are given in Tables 1 and 2, respectively.

#### 2.3.11. 7*α*, 8*β*, 12*β*, 18-Tetrahydroxy Pimar-15-en (11)

White crystalline needles, freely soluble in ethyl acetate and methanol; HR-ESI-MS [M-H]^−^ ion at *m/z* 337.2322 and molecular formula C_20_H_34_O_4._^13^C (100 MHz, MeOD) and ^1^H-NMR (400 MHz, MeOD) spectroscopic data are given in Tables 1 and 2, respectively.

#### 2.3.12. 3*β*, 8*β*, 11*β*, 12*α*, 18-Pentahydroxy Pimar-15-en (12)

White crystalline needles; freely soluble in ethyl acetate and methanol, HR-ESI-MS [M-H]- ion at *m/z* 353.2269 with molecular formula C_20_H_34_O_5_. ^13^C (100 MHz, MeOD) and ^1^H-NMR (400 MHz, MeOD) spectroscopic data are given in Tables 1 and 2, respectively.

#### 2.3.13. 12*β* Acetoxy, 8*β*, 3*β*, 18-Trihydroxy Pimar-15-en (13)

White crystalline needles; freely soluble in ethyl acetate and methanol, HR-ESI-MS [M-H]^−^ ion at *m/z* 379.2479 with molecular formula C_22_H_36_O_5_. ^13^C (100 MHz, MeOD) and ^1^H-NMR (400 MHz, MeOD) spectroscopic data are given in Tables 1 and 2, respectively.

#### 2.3.14. 3*β* Acetoxy, 8*β*, 12*β*, 18-Trihydroxy Pimar-15-en (14)

White crystalline needles; freely soluble in acetone; sparingly soluble in ethyl acetate and methanol, HR-ESI-MS [M-H] ^−^ ion at *m/z* 379.2473 with molecular formula C_22_H_36_O_5_. ^13^C (100 MHz, MeOD) and ^1^H-NMR (400 MHz, Acetone-*d*_*6*_) spectroscopic data are given in Tables 1 and 2, respectively.

### 2.4. Antimicrobial Assay

#### 2.4.1. Microbial ATCC Strains

To investigate the in vitro antimicrobial potency of isolated new compounds, Gram-positive bacteria *S. aureus (*ATCC 9144) and *S. epidermidis* (ATCC 12228) and Gram-negative bacteria (*P. aeruginosa* (ATCC 27853) and *E. coli* (ATCC 14948) were purchased from National Path Lab, Butwal, Nepal. All the ATCC strains were subcultured on different culture media and investigated for Gram staining and biochemical test. The detail of microbial analysis of all the strains is given on supplementary data (Table S1).

#### 2.4.2. Determination of Minimum Inhibitory Concentration (MIC)

The twofold serial broth microdilution technique was adopted to calculate the MIC values of new isopimarane diterpenoids, against four different test organisms. A total of 10 vials were labeled and sterilized, then 960 *µ*L of sterilized Mueller–Hinton Broth (MHB) was transferred into each vial. For the sample solution preparation, 25000 *µ*g/mL of stock solution was prepared in DMSO, subjected to serial dilution, using a 1 : 1 mixture of DMSO and water to prepare sample solutions of 10 different concentrations (25000 *µ*g/mL-48.8298125 mg/mL). After that, 40 *µ*L of sample solution was transferred into a corresponding vial containing 960 *µ*L of MHB, so that the final concentration of sample ranged from 1000 *µ*g/mL to 1.95 *µ*g/mL. Bacteria with an inoculum of about 1 × 10^5^ CFU/mL were loaded into each vial. For the preparation of microorganism inocula, broth culture was incubated for 12 h, and turbidity of the suspension was adjusted to the turbidity of 0.5 McFarland standards. One inoculated vial was used as a negative control, to ensure suitability broth for growth of microorganism growth. Also, 4% DMSO was tested as a blank control. Streptomycin sulfate and Vancomycin were considered as a positive control for Gram-negative and Gram-positive microorganisms, respectively. After the incubation of the sample containing broth media for 24 h at 37 ^0^C, the MIC value was determined. The MIC value was considered as the minimum concentration of compound that prevented the microorganism growth. The bacterial cell viability was determined by using 3(4,5 dimethylthiazol-2-yl)-2-5-diphenyl tetrazolium bromide (MTT) by incubating at 37°C for further 2 h and visual inspection of formazan formation [13].

## 3. Results and Discussion

### 3.1. Identification and Structure Elucidation of Noble Compounds

**Figure 1:**
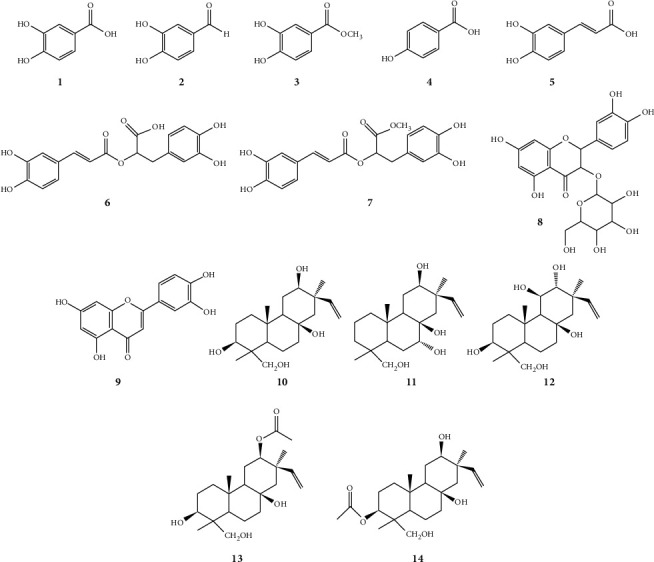
Chemical structures of compounds isolated from *Lycopus lucidus*.

The air dried aerial parts of *L. lucidus* were macerated with methanol at normal room temperature. The methanol extract was subjected to successive fractionation between hexane, thyl acetate, n-butanol, and water. Reverse phase open column chromatographic analysis of the ethyl acetate fraction enabled the isolation of five new pimarane diterpenoids: 3*β,* 8*β,* 12*β,* 18-tetrahydroxy pimar-15-en (**10**), 7*α,* 8*β,* 12*β*, 18-tetrahydroxy pimar-15-en (**11**), 3*β,* 8*β,* 11*β,* 12*α,* 18-pentahydroxy pimar-15-en (**12**), 12*β* acetoxy, 3*β,* 8*β*, 18-trihydroxy pimar-15-en (**13**), and 3*β* acetoxy, 8*β,* 12*β,* 18-trihydroxy pimar-15-en (**14**), along with nine known compounds. Structure of known compounds was confirmed as protocatechuic acid (**1**) [14], protocatechualdehyde (**2**) [14], methyl 3,4 dihydroxy benzoate (**3**) [15], para-hydroxy benzoic acid (**4**) [16], caffeic acid (**5**) [17], rosmarinic acid (**6**) [4], methyl rosmarinate (**7**) [4], quercetin 3-O-*β*-D-glucopyranoside (**8**) [18], and luteolin (**9**) [4], through the comparison of obtained physical and spectroscopic data with previous literature. Chemical structures of all the isolated compounds are depicted in Figure 1.

Compound **10** appeared as white crystalline needles with HR-ESI-MS [M-H]- ion at *m/z* 337.2354 and molecular formula C_20_H_34_O_4_. The ^1^H-NMR showed three olefin protons, twelve methylene groups, two oxygenated geminal methylene protons, two oxygenated methine protons, and two other normal methine protons, along with three methyl protons. The ^13^C-NMR exhibited 20 carbon signals consisting of two olefin carbon signals at *δ*_C_ 153.4 (C-15) and 108.9 (C-16), four oxygenated carbon signals at *δ*_C_ 75.6 (C-12), 74.9 (C-8-tertiary carbon), 68.5 (C-3), and 64.5 (C-18). Three methyl signals were detected at *δ*_C_ 33.7 (C-17), 25.0 (C-19), and 22.1 (C-20). Similarly, six other normal methylene carbons at *δ*_C_ 36.7 (C-1), 28.5 (C-2), 17.9 (C-6), 39.2 (C-7), 27.1 (C-11), and 45.9 (C-14) and two normal methine carbon signals at *δ*_C_ 43.9 (C-5) and 40.5 (C-9) were also recorded. Four carbon signals at *δ*_C_ 74.8 (C-8), 45.5 (C-4), 37.7 (C-13), and 33.9 (C-10) were found to be tertiary carbons through HMQC analysis. All protons were assigned to their corresponding carbons using HMQC experiment. Correlation of two oxygenated germinal methylene protons at *δ*_H_ 3.89 (1H, *d*, *J* = 12.0 Hz, H-18a) and 3.54 (1H, *d*, *J* = 12.0 Hz, H-18b) to carbon *δ*_C_ 64.5 (C-18) suggests presence of CH_2_OH group. Among three olefinic protons, two were found to be connected with carbon *δ*_C_ 153.4 (C-15). The presence of an ABX spin system in its ^1^H-NMR spectra due three vinyl protons of monosubstituted double bond at *d* 4.9 (dd, *J* = 1.2, 17.4 Hz, H-16a), *d* 4.79 (dd, *J* = 1.2, 17.4 Hz, H-16b), and *d* 5.78 (dd, *J* = 10.8, 17.2 Hz, H-15) along with the respective carbon resonances in the ^13^C-NMR spectra (*δ* 153.4 ppm, CH and *d* 108.9 ppm CH_2_) ensured the compound is pimar-15-en derivative [19, 20]. Analysis of the ^1^H-^1^H COSY plot of compound (**10)** suggested correlations (H1/H2/H3), (H5/H6/H7), (H9/H11/H12), and (H15/H16). In the HMBC data, correlations (C-11 and C-13)/H-12), (C-8/H-7), and ((C-18/(H-5 and H-3)) confirmed the positions of four hydroxyl groups; also carbon-proton correlations (C-17/H-15) and (C-20/H-1) ensured the exact position of C-17 and C-20 methyl group, respectively. Presence of hydroxyl group at C-8 was confirmed as it was tertiary oxygenated carbon. Relative configuration of compound was confirmed by ROESY analysis. Configuration of H-17 methyl group was found to be at *a* position through the observation of difference in chemical shift of *a* C-17 and *ß* C-17 in the literature [20–24]. Similarly, configuration of 8-hydroxyl group ensured to be at *ß* position by correlating with chemical shift of C-17 methyl group (*trans* configuration *δ*C-17 > 32 ppm, *cis* relationships *δ*C-17 < 25 ppm) [19–23]. From ROESY, relative configuration of H-3, H-5, H-9, H-12, and H-20 was confirmed from the correlation as follows: (H-17 to H-11b), (H-11b to H-12), which confirmed that hydroxyl group at C-12 should be at *β* position, correlation (H-11a to H-20) ensured *β* position of H-20, correlations (H-11b to H-9) and (H-9 to H-5) indicated *α* position of their proton, and finally correlations (H-9 to H-6a), (H-6a to H-18b), and (H-18b to H-3) ensured the *β* position of C-3 hydroxyl group. Therefore, chemical structure of compound **10** was established as 3*β,* 8*β,* 12*β*, 18-tetrahydroxy pimar-15-ene.

Compound **11** was obtained as white crystalline needles having HR-ESI-MS [M-H]- ion at *m/z* 337.2322 and molecular formula C_20_H_34_O_4._ It differs from compound 10 by presence of C-7-hydroxyl group instead of C-3. Hydroxylation at C-7 was confirmed by its chemical shift (*δ*_C_-74.1). Final structure of compound is confirmed as 7*α,* 8*β,* 12*β*, 18-tetrahydroxy pimar-15-ene.

Compound **12** was found to be white amorphous powder. Its molecular formula C_20_H_34_O_5_ was deduced by using HR-ESI-MS [M-H]- ion at *m/z* 353.2269. Spectral data of compound **12** was similar to compound **10** except new addition of hydroxyl group at C-11, which was ensured by its chemical shift value (*δ*_C_-73.1). ROESY ensured the chemical structure of compound **12** as 3*β,* 8*β,* 11*β,* 12*α*, 18-pentahydroxy pimar-15-ene.

Compound **13** was white in color with crystalline needles. Its molecular formula C_22_H_36_O_5_ was deduced by using HR-ESI-MS [M-H]- ion at *m/z* 379.2479. Spectral data of compound **13** was different from compound **10** by addition of new methyl proton as an acetyl moiety at *δ*_H_ 2.06 (3H,s, H-22) along with one new tertiary carbonyl carbons signal at *δ*_C_ 172.2 (-CO, C-21) and one methyl carbon at *δ*_H_ 22.4 (C-22). Increment of mass of compound **13** by 42 units [-COCH_3_-H^+^] also confirmed the addition of one acetyl moiety. Position of acetyl group was ensured accurately by other 2D-NMR analysis. In the HMBC data, carbon–proton correlations (C-21/H-22) illustrated attachment of the methyl group with carbonyl carbon. Correlation (C-12/H-21) verified the position of acetyl group. ROESY analysis illustrated the chemical name of compound 13 as 12*β* acetoxy, 8*β,* 3*β*, 18-trihydroxy pimar-15-ene.

Compound **14** also was white crystalline needles having similar molecular formula, mass fragmentation, and spectral data with compound **13**. It differed from compound **13** only by position of acetyl moiety. Chemical shift of C-3 (*δ*_H_ 65.9) gives an idea about its oxygenation. From the HMBC data, carbon–proton correlations (C-21/H-22) illustrated that methyl group is attached with carbonyl carbon and another association (C-22/H-18) suggested the location of acetyl group at C-3. Chemical name compound was deduced as 3*β* acetoxy, 8*β,* 12*β*, 18-trihydroxy pimar-15-ene. The ^13^C and ^1^H-NMR spectroscopic data of compound 10–14 are given in Tables 1 and 2, respectively.

### 3.2. Antimicrobial Activity of Isolated Pimarane Diterpenoids

The isolated new pimarane diterpenoids were examined for their antimicrobial potency in response to Gram-positive bacteria *S. epidermidis* (ATCC 12228) and *S. aureus* (ATCC 9144) and Gram-negative bacteria *P. aeruginosa* (ATCC 27853) and *E. coli* (ATCC 14948), using a twofold serial broth dilution technique and MIC was evaluated. As shown in Table 3, the most significant compound was **10**, which manifested MIC values of 15.62 and 31.25 *µ*g/mL against *S. epidermidis* and *S. aureus*, respectively. Also, this compound was effective against *P. aeruginosa* and *E. coli* with MICs of 62.5 *µ*g/mL and 125 *µ*g/mL, respectively. It is to be noted that compounds 13, 14, and 15 were ineffective against both Gram-negative strains at examined concentrations.

In the search for natural products as effective antimicrobial agents, numerous investigations have proved the promising bactericidal effect revealed by diterpenoids. However, very limited researches have been reported for the antibacterial potency of pimarane diterpenoids [25]. It has been reported that the presence of a decalin ring system in pimarane diterpenoids fascinates its penetration into the lipophilic cell membrane of bacteria to induce bacterial lysis. Furthermore, an appropriately positioned hydrophilic functional group (hydrogen bond donor group; HBD) is capable of interacting with phosphorylated groups of the bacterial cell membrane [26]. In this study, MIC values of two isomers, i.e., compounds **10** and **11**, were slightly different. The structural difference between these compounds is only the position of one hydroxyl group (3^rd^ position OH group in compound **10** is shifted to 7^th^ position in compound **11**). Although the exact structure-activity relationship is not known, this may signify that a change in position of the same functional group may also alter the antibacterial effect. On the other hand, the antibacterial activity of compounds 13 and 14 was reported to be reduced, in which -OH group was replaced by an electron-withdrawing acetyl group. In a previous study, 3- and 19-hydroxyl groups of isopimarane compounds were acetylated to investigate the role of substituents on antimicrobial activity. The results indicated that if acetylation occurs in the 3-hydroxyl position or both 19- and 3-hydroxyl groups, the antibacterial activity gets reduced significantly [27]. Hence, our study also revealed a similar result. Furthermore, this study suggested that a slight variation in the position and/or nature of the oxygenated moiety can result in a substantial change in antibacterial activity. Similar results were also found in previous studies, regarding the biological activities of other pimarane diterpenoids [25, 28, 29].

## 4. Conclusion

Five new pimarane diterpenoids along with nine known compounds (polyphenol and flavonoids) were isolated from the ethyl acetate fraction of *L. lucidus* and their chemical structures were elucidated completely through instrumental data including 2D-NMR.

Among them, compound **6** (rosmarinic acid) was isolated in the largest amount. Compound **2** (protocatechualdehyde) and compound **4** (methyl 3, 4, dihydroxy benzoate) were isolated for the first time from this plant. Besides, antibacterial activity screening for newly isolated compounds showed that pimarane diterpenoids are more sensitive towards Gram-positive bacteria. Among the isolated new diterpenoids, compounds **10** and **11** were found to be most effective with 15.62 *µ*g/mL and 31.25 *µ*g/mL MICs values, respectively, against *S. epidermidis*. In addition, this screening showed that small changes in position and/or nature of the oxygenated functional group in diterpenoids can result in a significant variation in antibacterial activity.

## Figures and Tables

**Table 1:** ^13^C-NMR spectroscopic data of compounds 10–14 at 100 MHz.

C	*δ*_C_-10	*δ*_C_-11	*δ*_C_-12	*δ*_C_-13	*δ*_C_-14
1	36.7	37.7	37.7	36.6	35.1
2	28.5	20.7	28.4	28.4	26.7
3	68.5	43.5	68.4	68.1	65.9
4	46.5	42.0	48.3	46.2	45.1
5	43.9	44.4	43.4	45.2	43.3
6	17.9	25.9	17.6	17.8	19.3
7	39.2	75.1	39.0	39.0	39.3
8	74.9	74.12	74.8	73.4	75.9
9	40.5	48.0	44.2	41.6	39.8
10	33.9	34.0	34.6	33.8	43.4
11	27.1	27.4	73.1	24.2	26.3
12	75.6	75.7	77.5	77.8	75.0
13	37.7	41.5	38.34	37.6	37.33
14	45.9	39.4	46.1	45.6	46.9
15	153.4	149.7	153.4	153.0	153.4
16	108.9	111.8	109.0	109.1	108.3
17-CH_3_	33.7	33.9	33.4	33.6	34.1
18	64.6	64.5	62.3	64.4	65.5
19-CH_3_	25.0	26.1	25.1	25.0	24.6
20-CH_3_	22.1	22.29	24.5	21.8	22.9
21-CO-	—	—	—	172.2	171.1
22- OCH_3_	—	—	—	21.4	21.1

Note: compounds **10**, **11**, **12**, and **13** were analyzed in methanol-*d*_*4*,_ Compound **14** was analyzed in acetone-*d*_*6*._

**Table 2:** ^1^H-NMR spectroscopic data of compounds 10–14 at 400 MHz.

C	^1^H-10	^1^H-11	^1^H-12	^1^H-13	^1^H-14
1	1.70 (1H, m, H-1a) 1.22 (1H, m, H-1b)	1.51 (1H, m, H-1a) 0.93 (1H, m, H-1b)	1.83 (1H, m, H-1a) 1.14 (1H, m, H-1b)	1.70 (1H, m, H-1a) 1.20 (1H, m, H-1b)	1.81 (1H, m, H-1a) 1.11 (1H, m, H-1b)
2	1.94 (1H, m, H-2a) 1.50 (1H, m, H-2b)	1.60 (1H, m, H-2a) 1.43 (1H, m, H-2b)	1.97 (1H, m, H-2a) 1.56 (1H, m, H-2b)	1.96 (1H, m, H-2a) 1.56 (1H, m, H-2b)	1.83 (1H, m, H-2a) 1.50 (1H, m, H-2b)
3	3.39–3.42 (1H, m, H-3)	1.46 (1H, m, H-3a 1.27 (1H, m, H-3b)	3.59 (1H, t H-3)	3.43 (1H, m, H-3)	4.1 (1H,t, H-3)
5	2.07 (1H, dd, 2.0, 12.6 Hz, H-5)	1.92 (1H, m, H-5)	2.015 (1H, dd,2.0, 12.6 Hz, H-5)	2.03 (1H, dd, 2.0, 12.6 Hz, H-5)	1.96 (1H, m, H-9)
6	1.78 (1H, m, H-6a) 1.45 (1H, m, H-6b)	2.04 (1H, m, H-6a) 1.54 (1H, m, H-6b)	1.76 (1H, m, H-6a) 1.48 (1H, m, H-6b)	1.76 (1H, m, H-6a) 1.45 (1H, m, H-6	2.00 (1H, m- H6a) 1.70 (1H, m, H-6b)
7	1.42 (2H, m, H-7)	3.57 (1H, t, H-7)	1.43 (2H, m, H-7)	1.50 (2H, m, H-7)	1.42 (1H, m, H-7a) 1.25 (1H, m, H-7b)
9	1.91 (1H, m, H-9)	1.65 (1H, m, H-9)	2.042, (1H, d, 2.8 Hz, H-9)	1.84 (1H, m, H-9)	2.15 (1H, dd, 2.0, 13.2 Hz, H-5)
11	2.38 (1H, td, 2.8, 2.4, 13.2 Hz, H-11a) 1.56 (1H, m, H-11b)	2.41 (1H, td, 2.8, 2.4, 13.2 Hz, H-11a) 1.56 (1H, m-H11b)	4.1 (1H, t, H-11)	2.39(1H, td, 2.8, 2.4, 13.2 Hz, H-11a 1.66 (1H, m, H-11b)	1.93 (1H, m, H-11a) 1.53 (1H, m, H-11b)
12	3.39–3.42 (1H, m, H-12)	3.48 (1H, m, H-12)	3.28 (1H, m, H-12)	4.62 (1H,t, H-12)	3.49 (1H, m, H-12)
14	2.015 (1H, d,14.4 Hz, H-14a) 1.19 (1H, m, H-14b)	2.347(1H, d, 14.4 Hz, H-14a) 1.098 (1H, dd, 0.8, 14.4 Hz, H-14b)	1.93 (1H, m, H-14a) 1.26 (1H, m, H-14b)	1.63 (1H, m, H-14a) 1.23 (1H, m, H-14b)	2.03 (1H, m, H-14a) 1.15 (1H, m, H-14b)
15	5.78 (1H, dd, 10.8, 17.2 Hz, H-15)	5.91 (1H, dd, 10.8, 17.2 Hz, H-15)	5.76 (1H, dd, 10.8, 17.2 HzH-15)	5.75 (1H, dd, 10.8, 17.2 Hz, H-15)	5.76 (1H, dd, 10.8, 17.2 Hz, H-15)
16	4.90 (1H, dd, 1.2, 17.4 Hz, H-16a) 4.79 (1H, dd, 1.2, 17.4 Hz, H-16b)	5.00 (1H, dd, 1.2, 14.2 Hz, H-16a) 4.97 (1H, dd, 1.2, 7.6 Hz, H-16b)	4.88 (1H, dd, 1.2, 17.4 Hz, H-16a) 4.79 (1H, dd, 1.2, 17.4 Hz, H-16b)	4.86 (1H, dd, 1.2,17.4 Hz, H-16a) 4.80 (1H, dd, 1.2, 17.4 Hz, H-16b)	4.84 (1H, dd, 1.2, 17.4 Hz, H-16a) 4.74 (1H, dd, 1.2, 17.4 Hz, H-16b)
17-CH_3_	0.92 (3H, s, H-17)	0.912 (3H, s, H-17)	1.01 (3H, s, H-17)	0.84 (3H, s, H-17)	0.88 (3H, s, H-17)
18	3.89 (1H, d, 12.0 Hz, H-18a) 3.54 (1H, d, 12.0 Hz, H-18b)	4.50 (1H, d, 12 Hz, H-18a) 3.487 (1H, d, 12 Hz, H-18b)	3.85 (1H, d,12.0 Hz, H-18a) 3.54 (1H, d,12.0 Hz, H-18b)	3.94 (1H, d,12.0 Hz, H-18a) 3.56 (1H, d, 12.0 Hz, H-18b)	5.08 (1H, d, 12.0 Hz, H-18a) 4.52 (1H, d, 12.0 Hz, H-18b)
19-CH_3_	1.24 (3H, s, H-19)	1.26 (3H, s, H-19)	1.24 (3H, s H-19)	1.24 (3H,s, H-19)	1.23 (3H, s, H-19)
20-CH_3_	1.01 (3H, s, H-20)	1.01(3H,s, H-20)	1.25 (3H, s, H-20)	1.00 (3H, s, H-20)	0.89 (3H, s, H-20)
22-OCH_3_				2.06 (3H, s, H-22)	1.98 (3H, s, H-22)

**Table 3:** Results of the in vitro antibacterial activity (MIC) of compounds **10–14** against selected Gram-positive and Gram-negative bacteria.

Microorganisms	Minimum inhibitory concentration (*µ*g/mL)						
	10	11	12	13	14	Van	Strep
S. *aureu*s (ATCC 9144)	31.25	31.25	62.5	125	125	0.488	—
*S. epidermidis* (ATCC 12228)	15.62	31.25	125	250	250	0.488	—
*P. aeruginosa* (ATCC 27853)	62.5	62.5	125	—	—	—	7.812
*E. coli* (ATCC 14948)	125	250	—	—	—	—	7.812

Van: Vancomycin hydrochloride; Strep: streptomycin sulfate; negative control: 4% DMSO solution did not suppress the growth of the tested bacteria.

## Data Availability

All the data used to support the result of this research are available from Jitendra Pandey.

## Supplementary Materials

Supplementary Materials The spectroscopic data used to support the findings of our research are available in Supplementary Figures S1–S35 and Table S1. .
