# Supplementary material for: Pimarane Diterpenoids from Aerial Parts of Lycopus lucidus and Their Antimicrobial Activity
Source: Evid Based Complement Alternat Med. 2022 Feb 4;2022:5178880. doi: 10.1155/2022/5178880 (PMC8837446; doi:10.1155/2022/5178880)
Supplement: Supplementary Materials — The spectroscopic data used to support the findings of our research are available in Supplementary Figures S1–S35 and Table S1. . [file 5178880.f1.docx]

Supplementary Materials


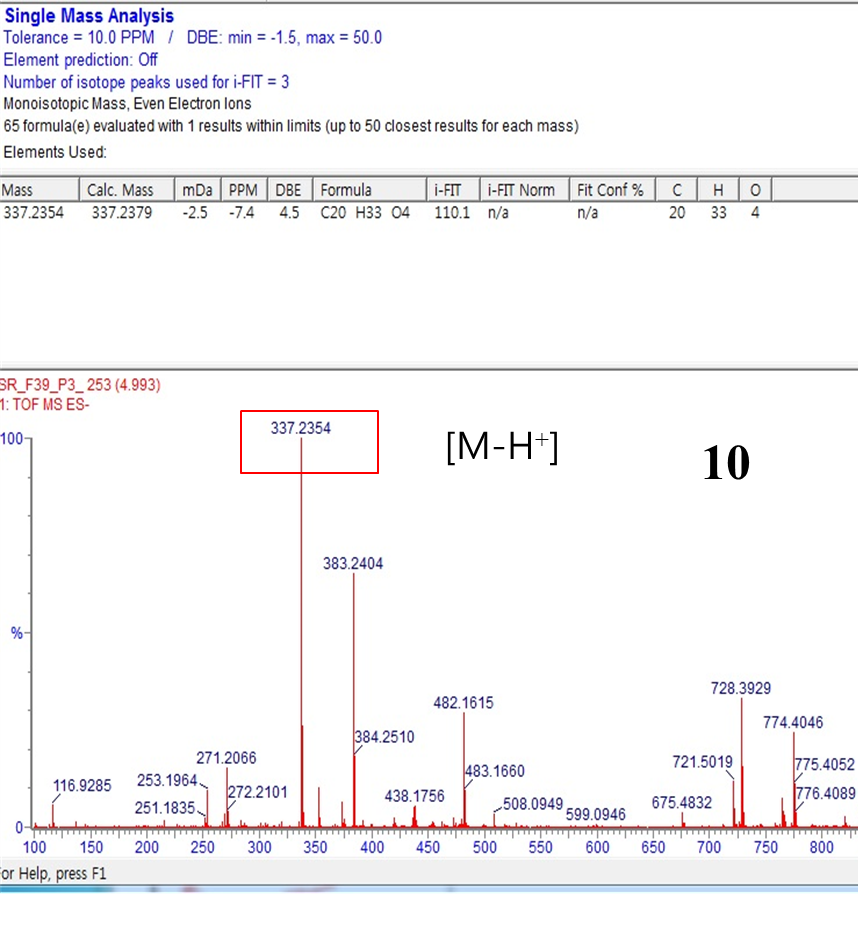


FIGURE S1: HR-MS [M-H^+^] of Compound 10


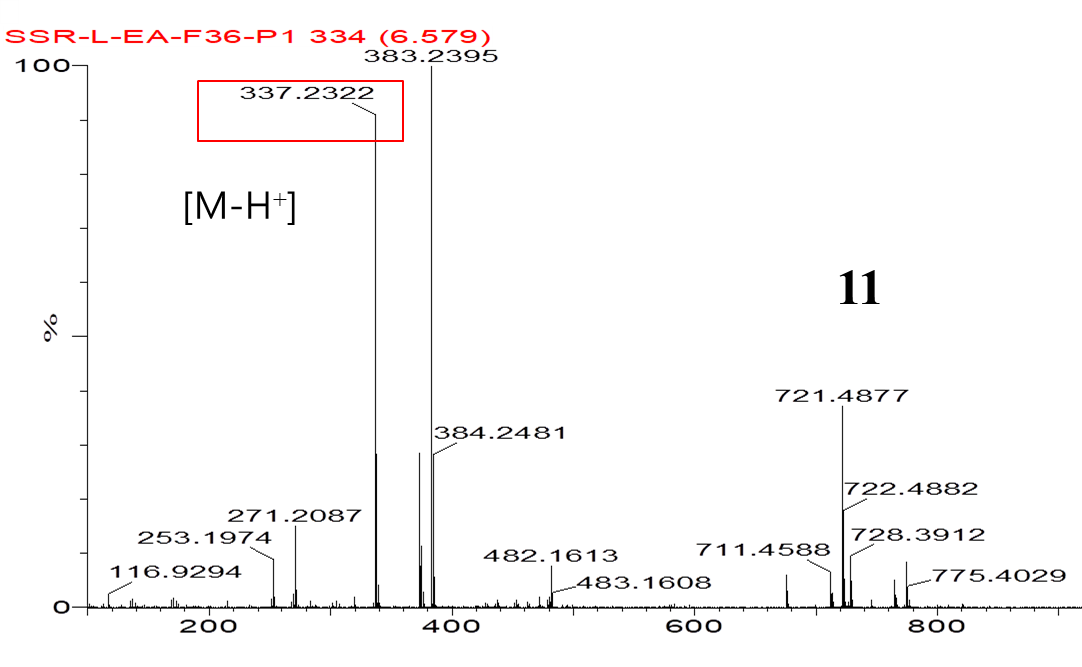


FIGURE S2: HR-MS [M-H+] of Compound 11


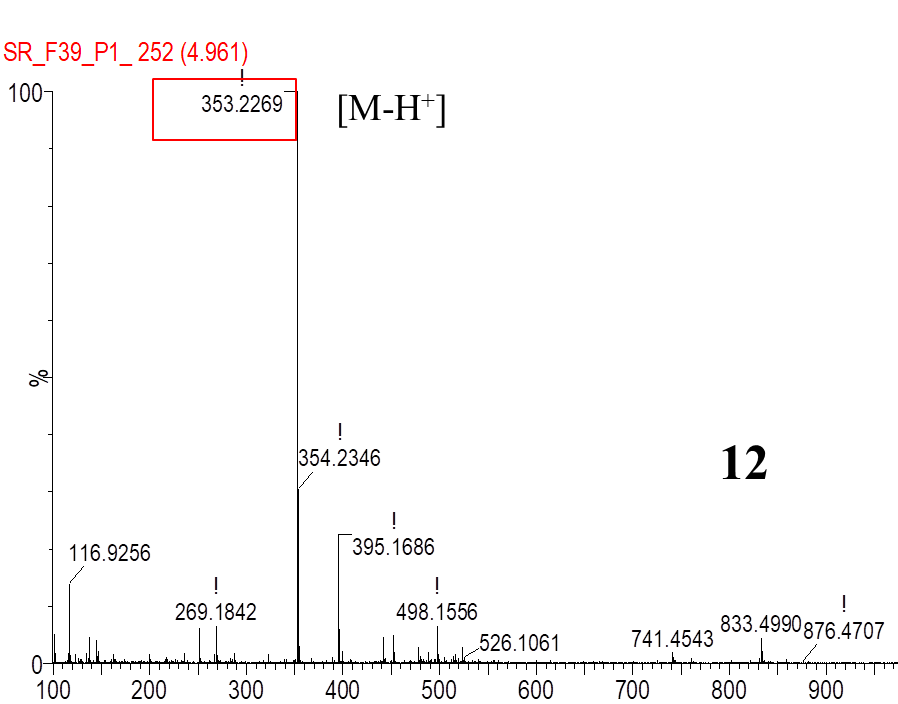


FIGURE S3: HR-MS [M-H^+^] of Compound 12


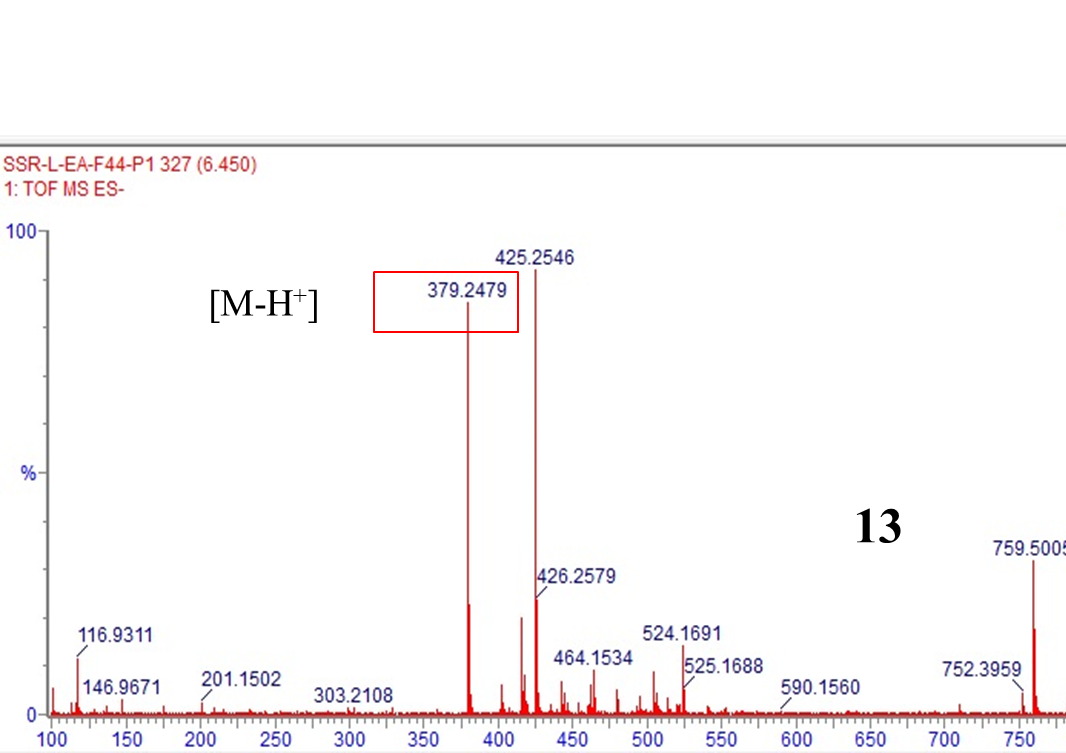


FIGURE S4: HR-MS [M-H^+^] of Compound 13


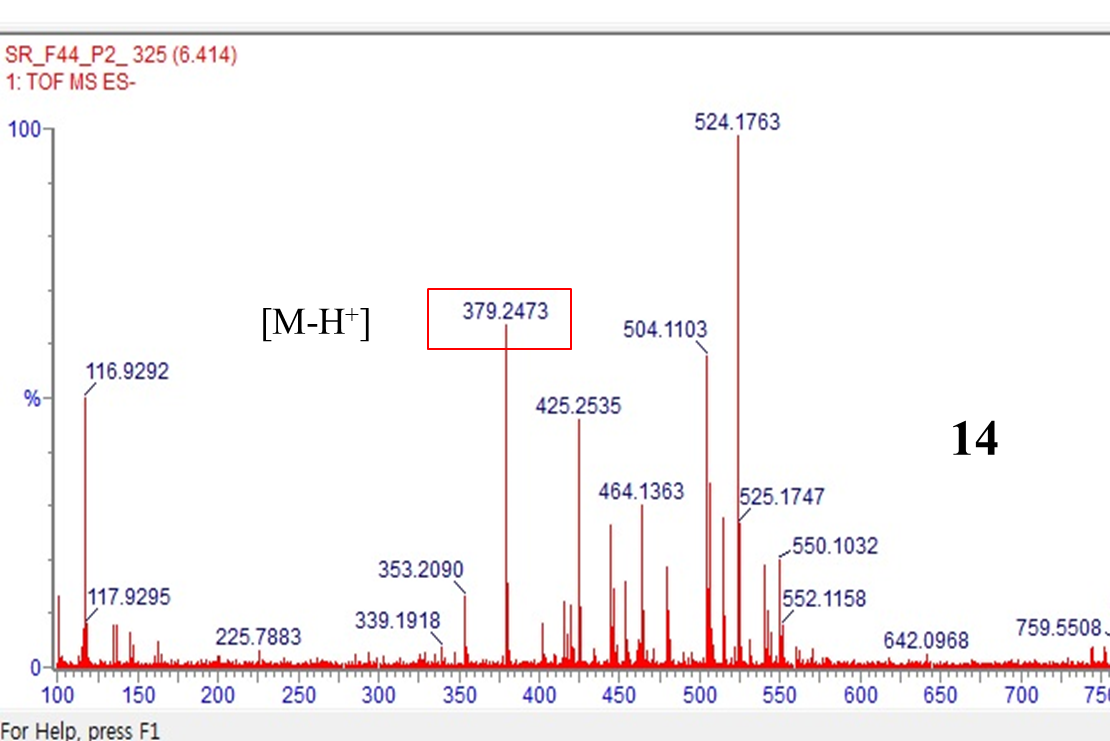


FIGURE S5. Figure S4: HR-MS [M-H^+^] of Compound 14

**
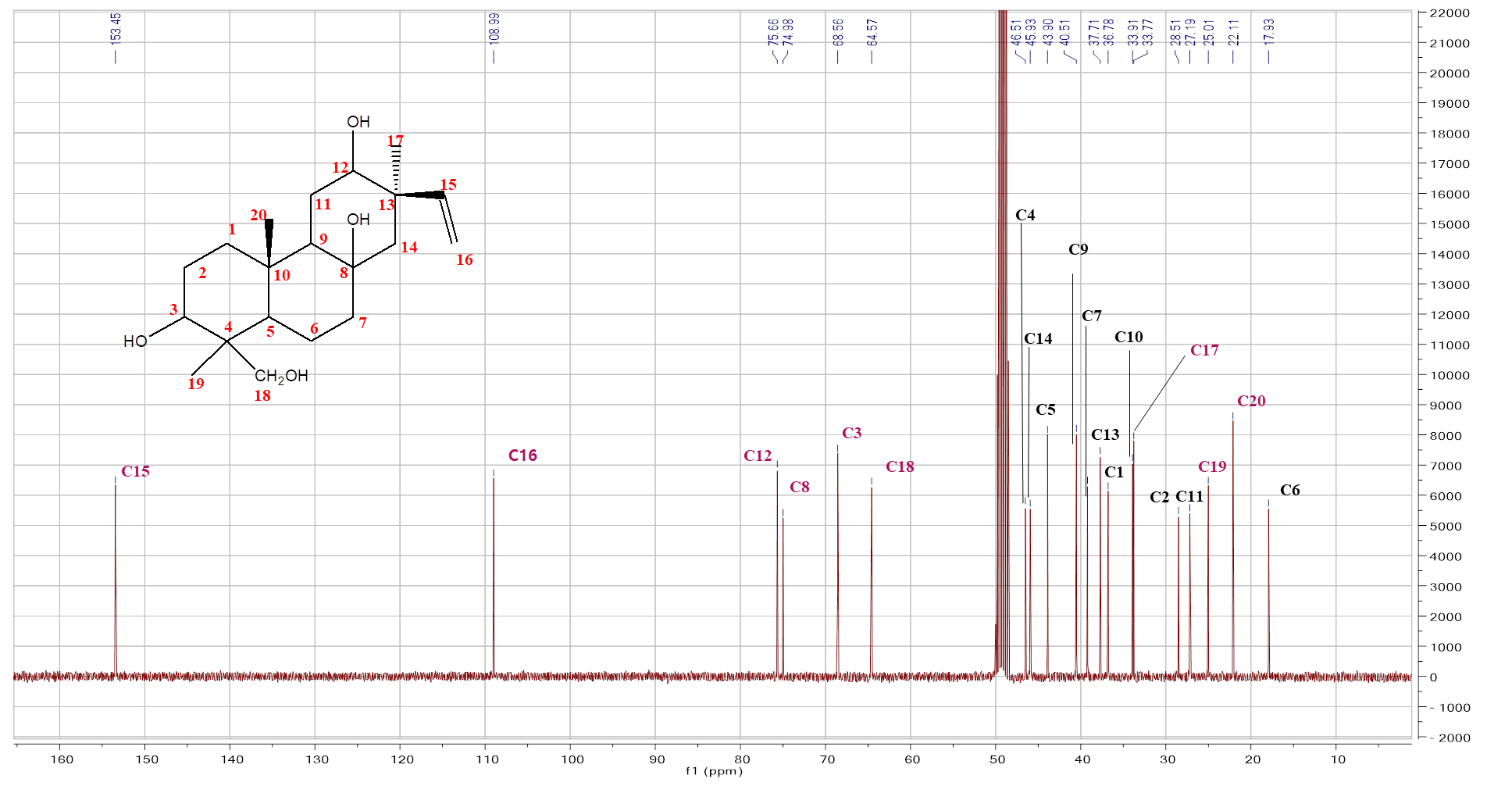
**

FIGURE S6: ^13^C NMR of Compound **10** ( 100 MHz, Methanol- *d_4_*)

**
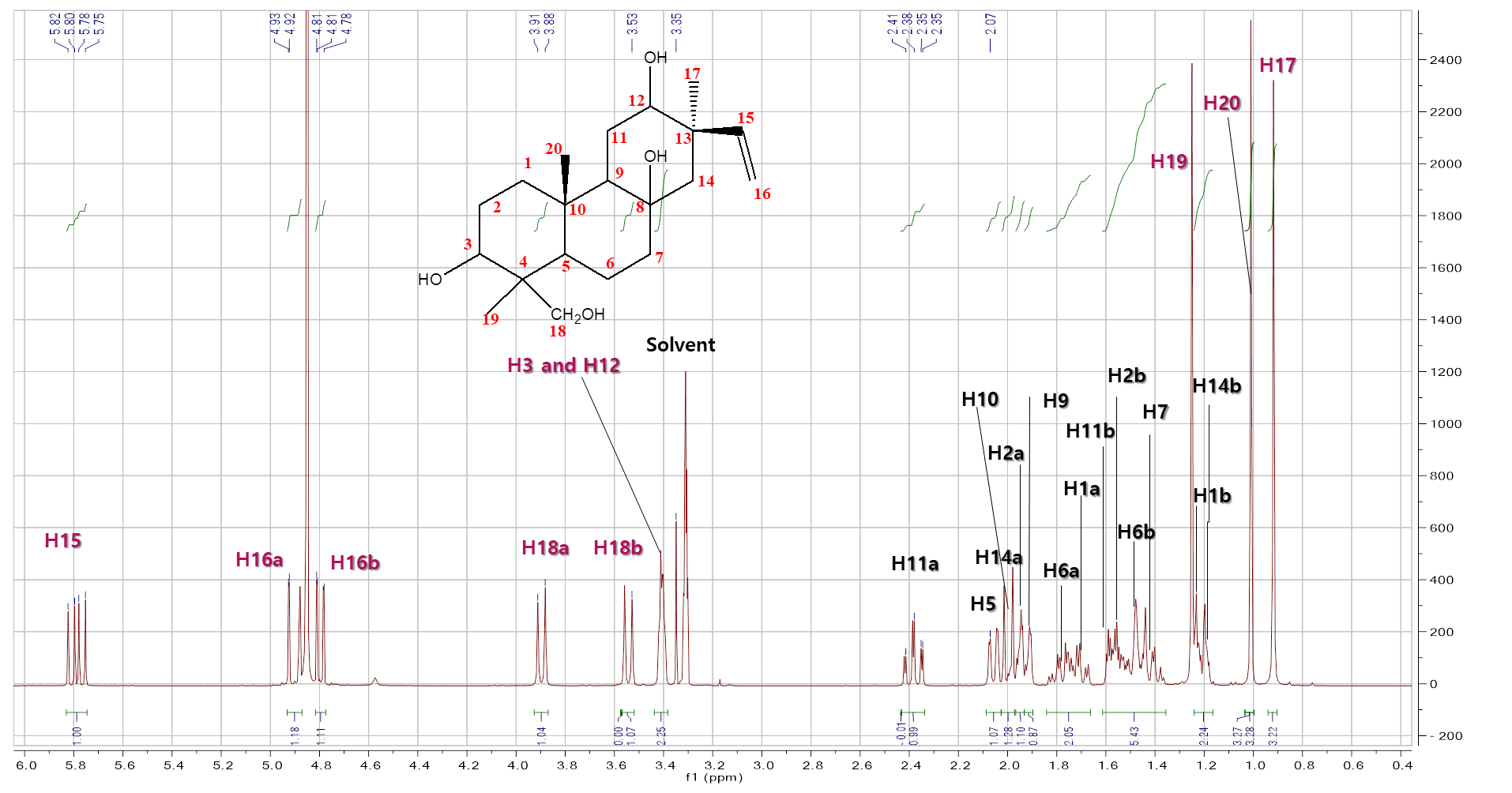
**

FIGURE S7: ^1^H NMR of Compound **10** (400 MHz, Methanol- *d*_4_)

**
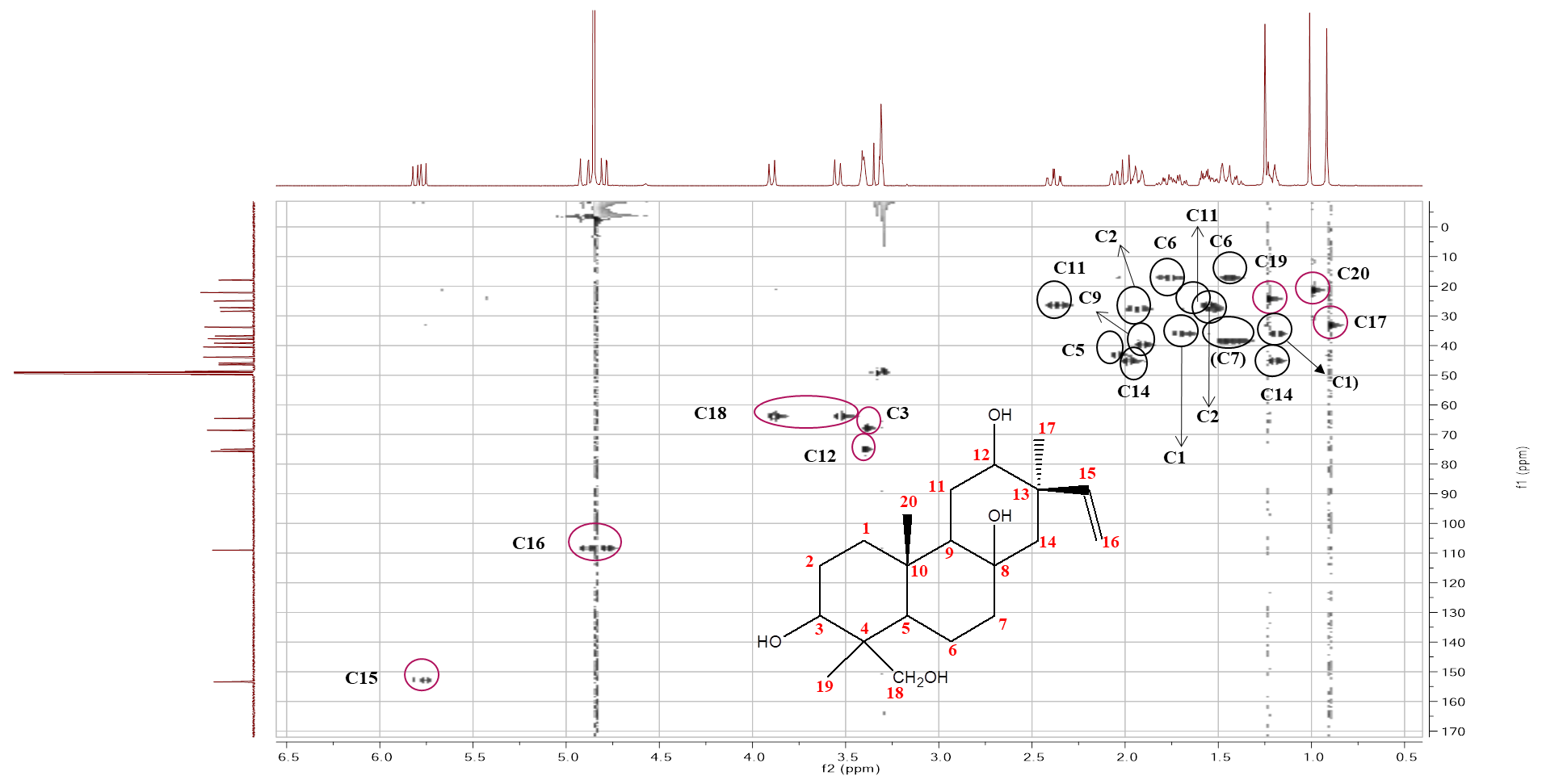
**

FIGURE S8**:** HMQC spectrum of compound **10** (Methanol- *d*_4_)

**
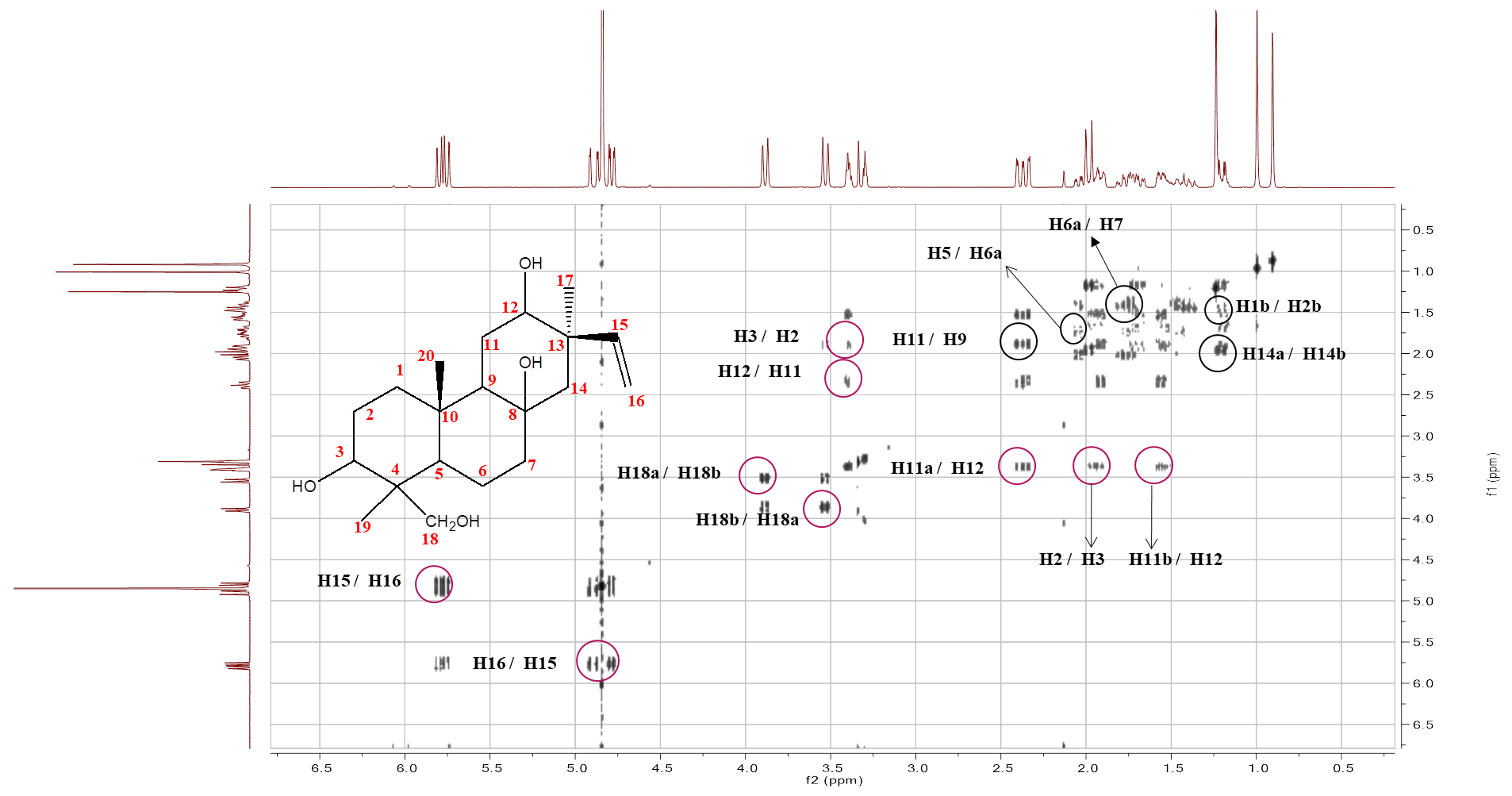
**

FIGURE S9: COSY spectrum of compound **10** (Methanol- *d*_4_)


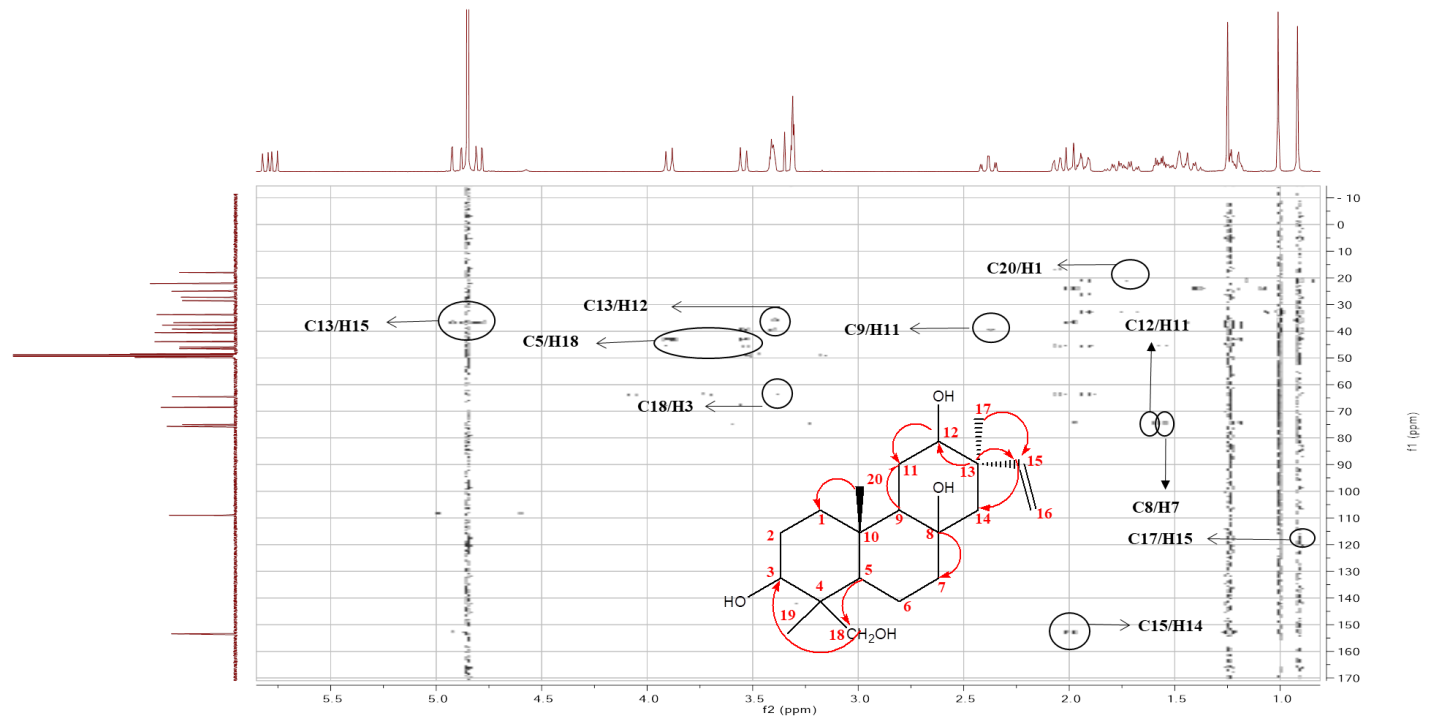


FIGURE S10: HMBC spectrum of compound **10** (Methanol- *d*_4_)


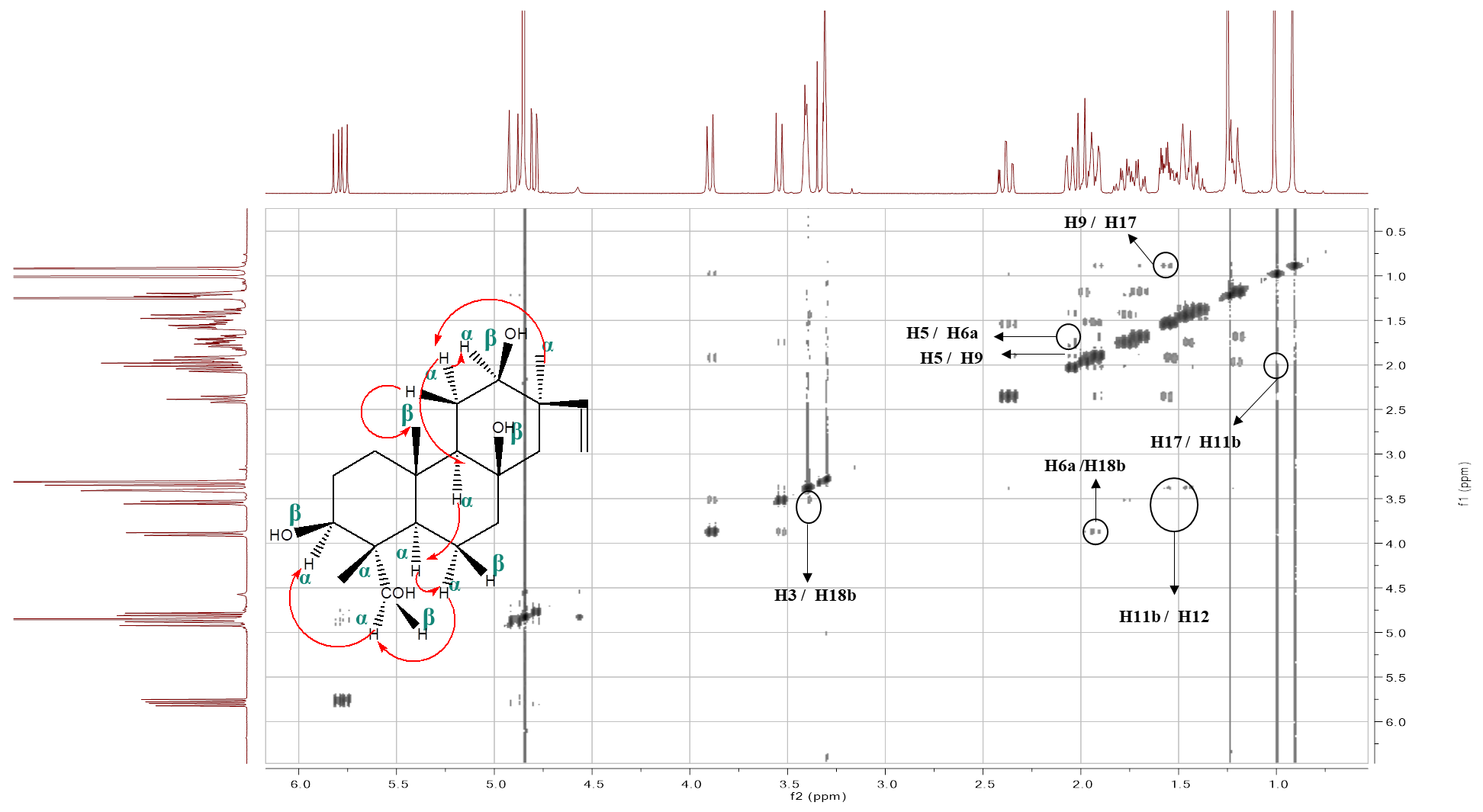


FIGURE S11: ROESY spectrum of compound **10** (Methanol- *d*_4_)


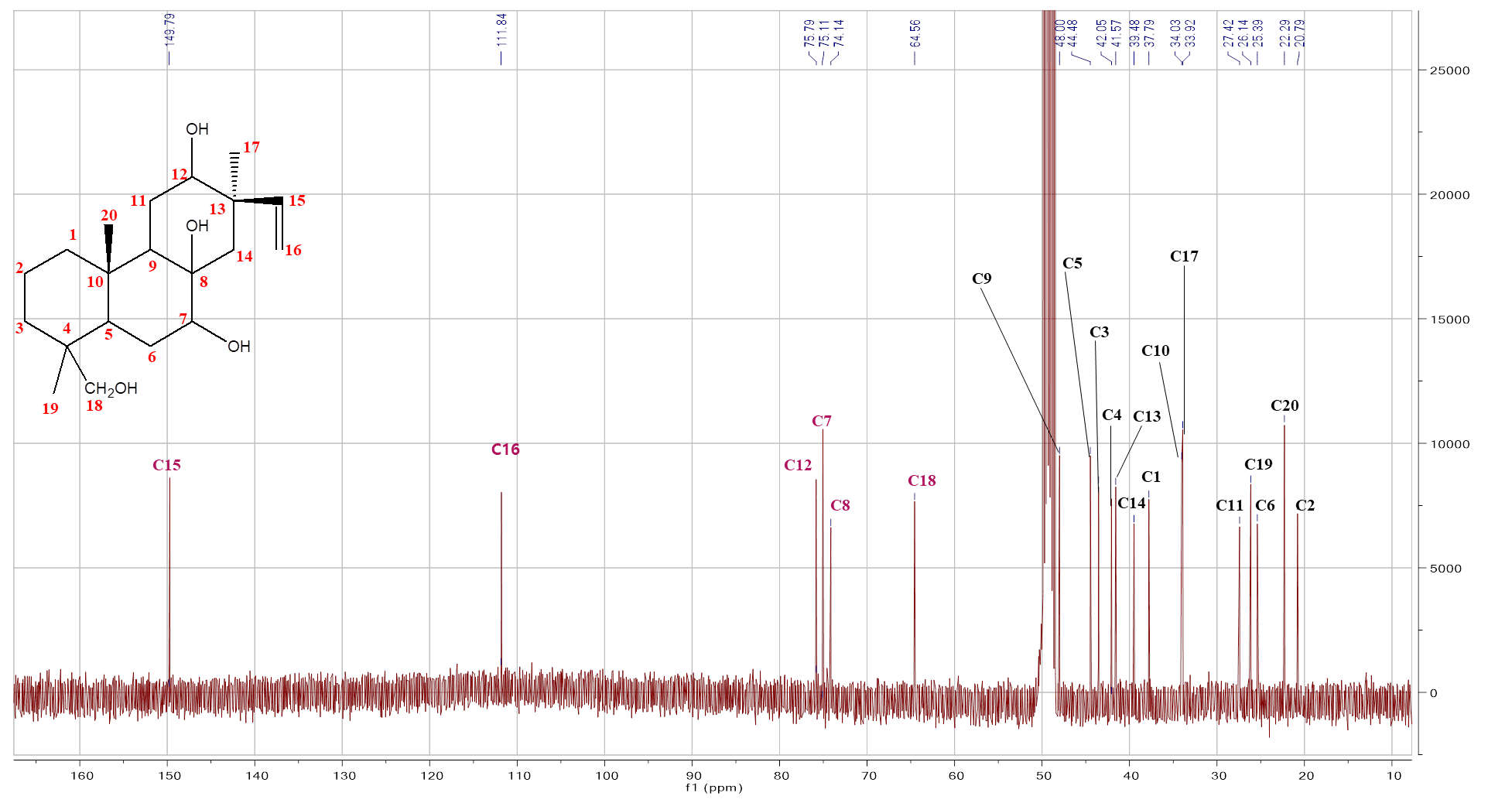


FIGURE S12: ^13^C NMR of Compound **11** (100 MHz, Methanol- *d*_4_)


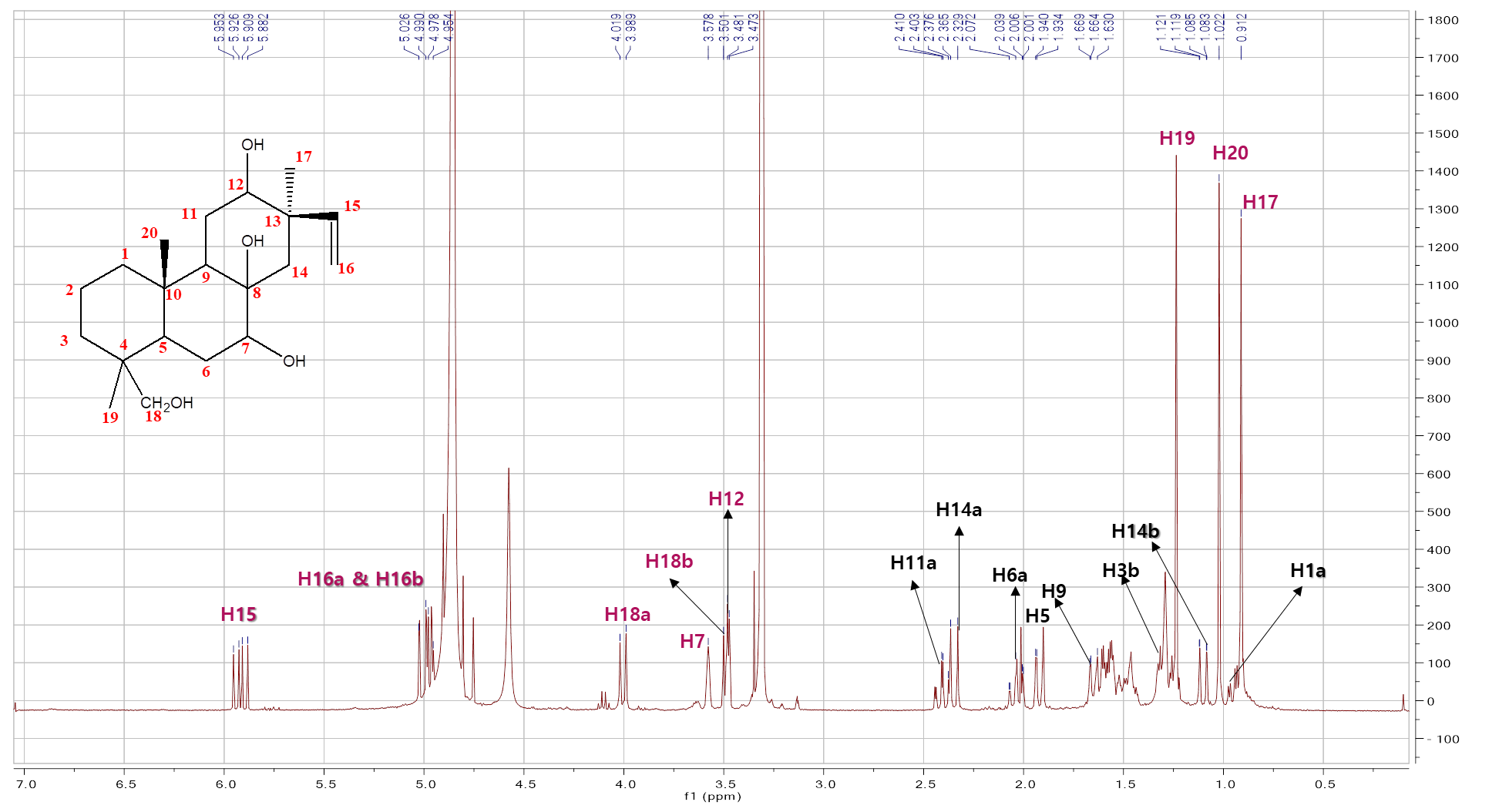


Figure S13: ^1^H NMR of Compound **11** (400 MHz, Methanol- *d*_4_)


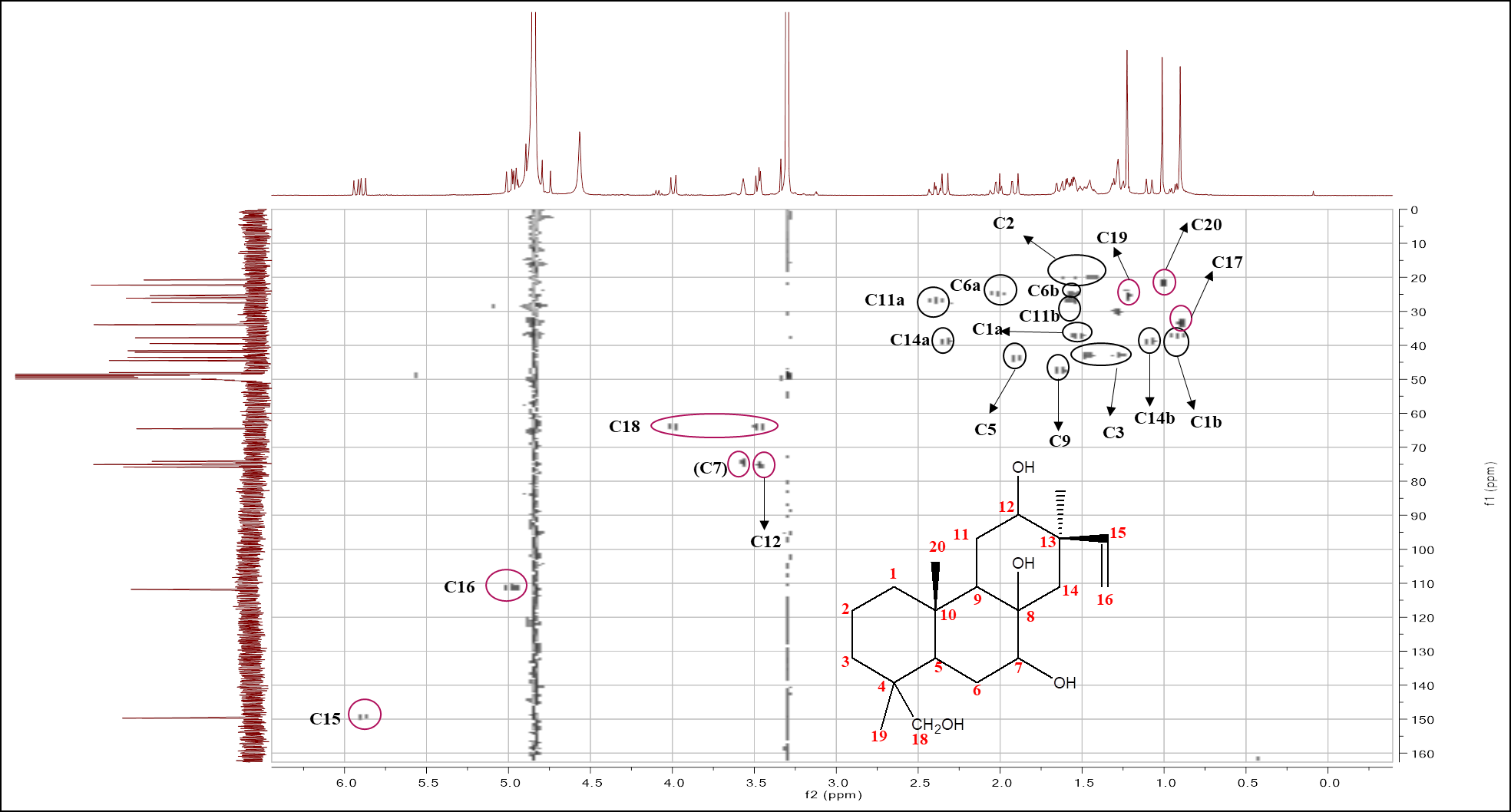


FIGURE S14: HMQC spectrum of compound **11** (Methanol- *d*_4_)


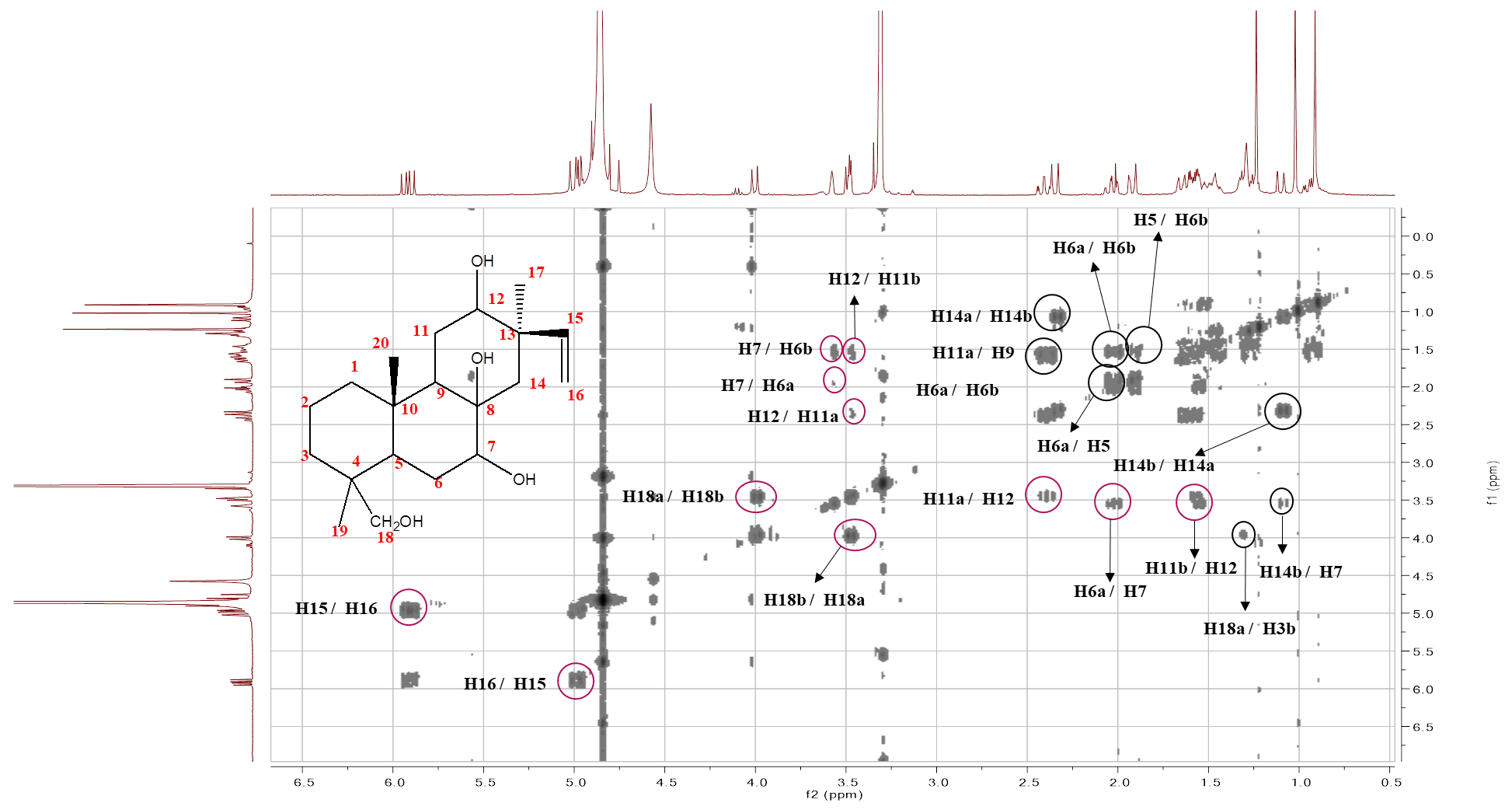
FIGURE 15: COSY spectrum of compound **11** (Methanol- *d*_4_)


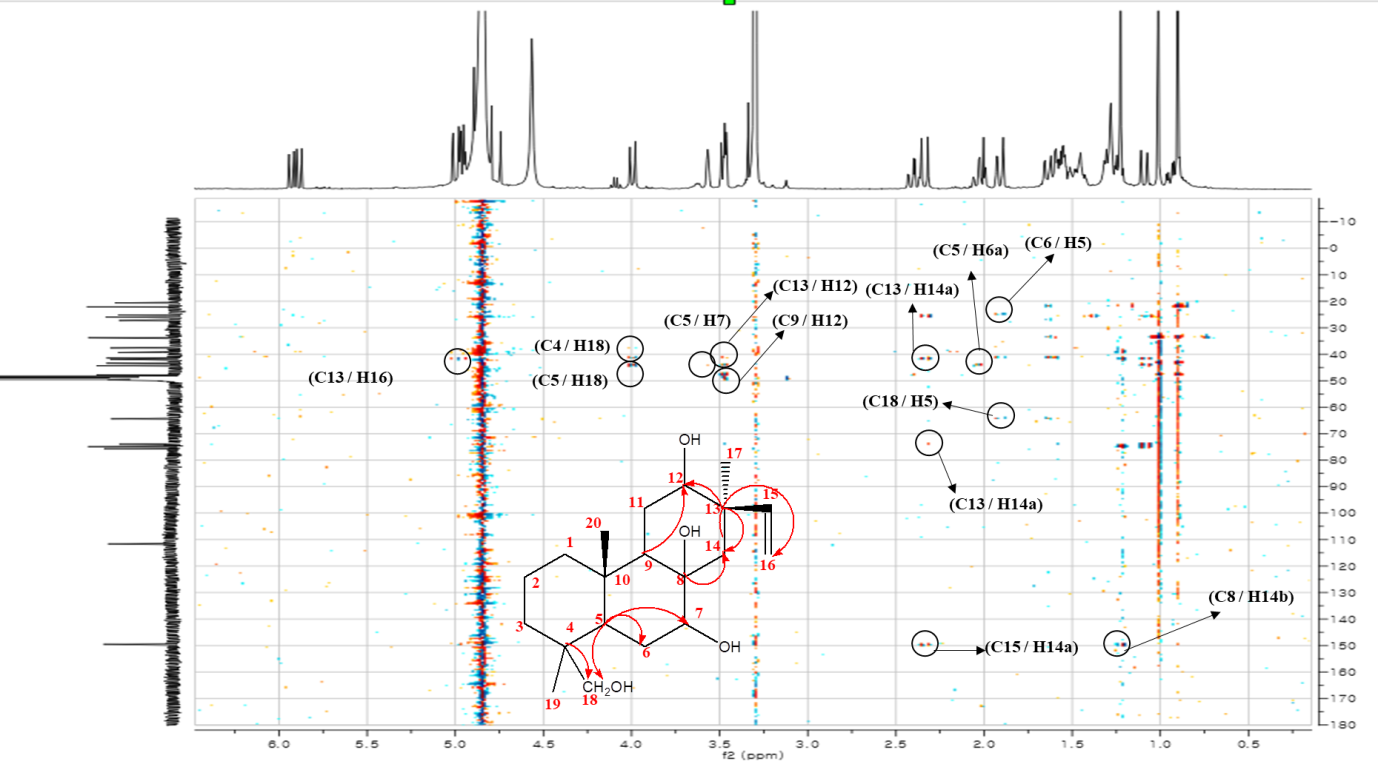
FIGURE 16: HMBC spectrum Compound **11**(Methanol-d4)


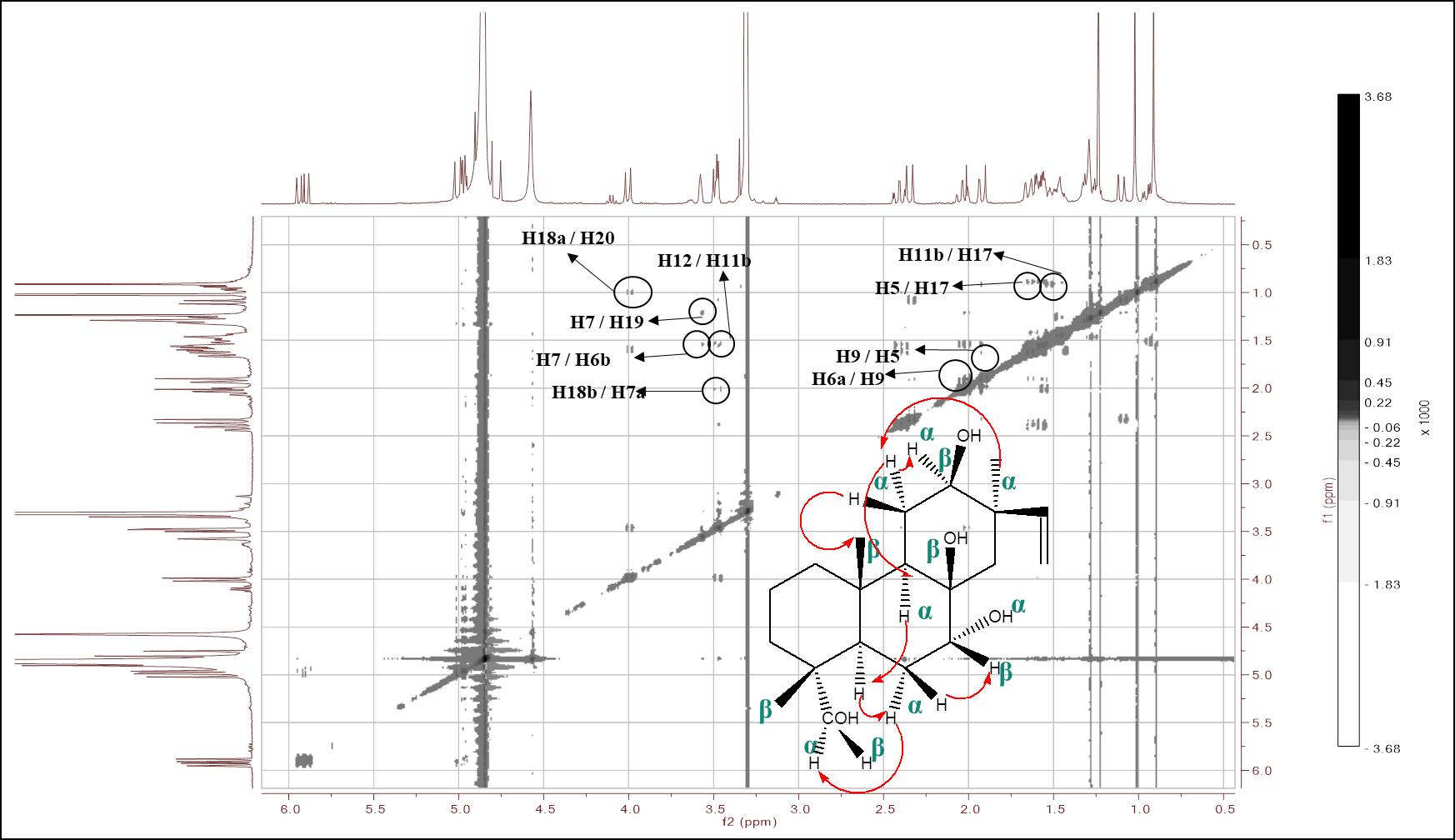


FIGURE S17: ROESY spectrum of compound **11** (Methanol- *d*_4_)


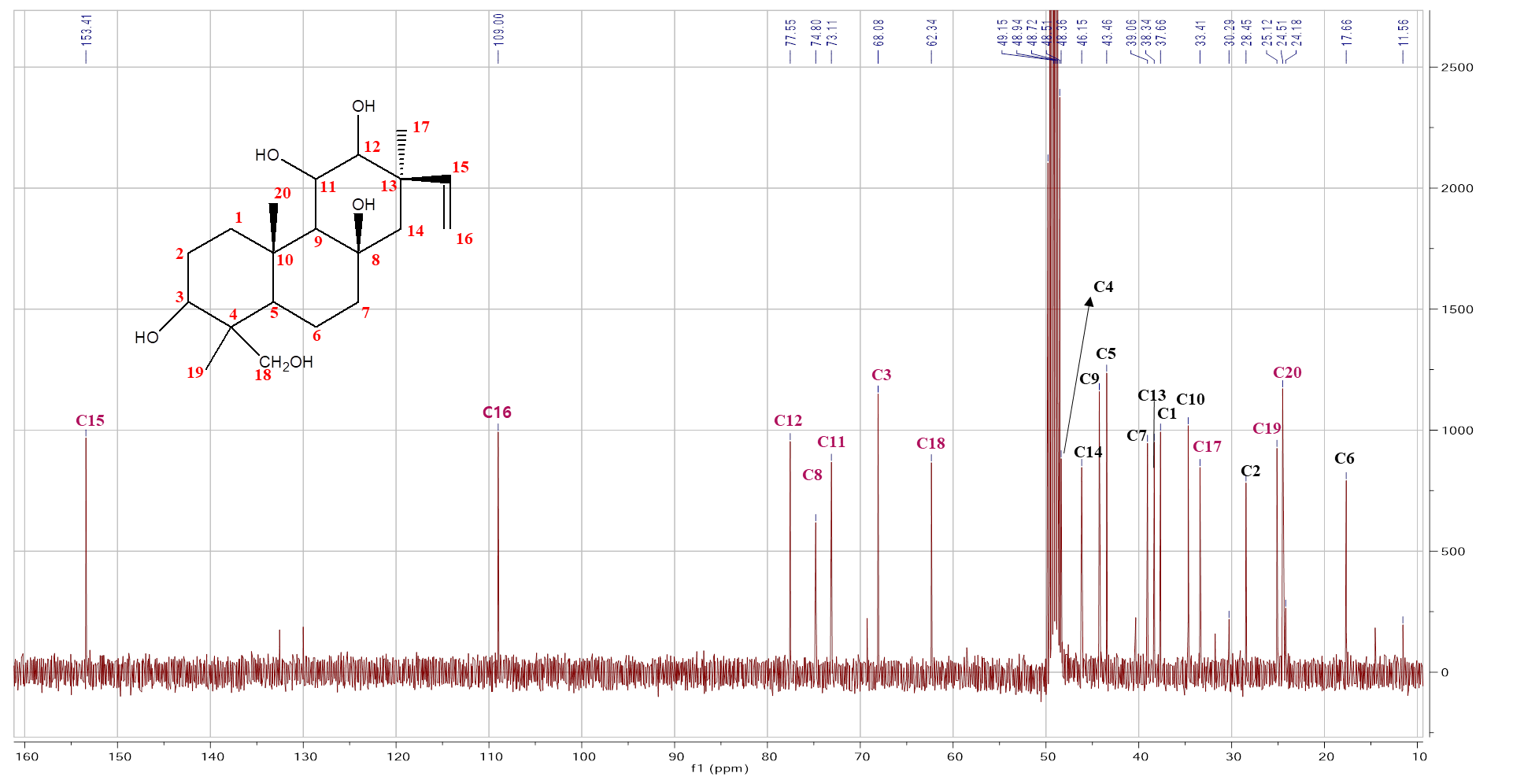


FIGURE S18: ^13^C NMR of Compound **12** (100 MHz, Methanol- *d*_4_)


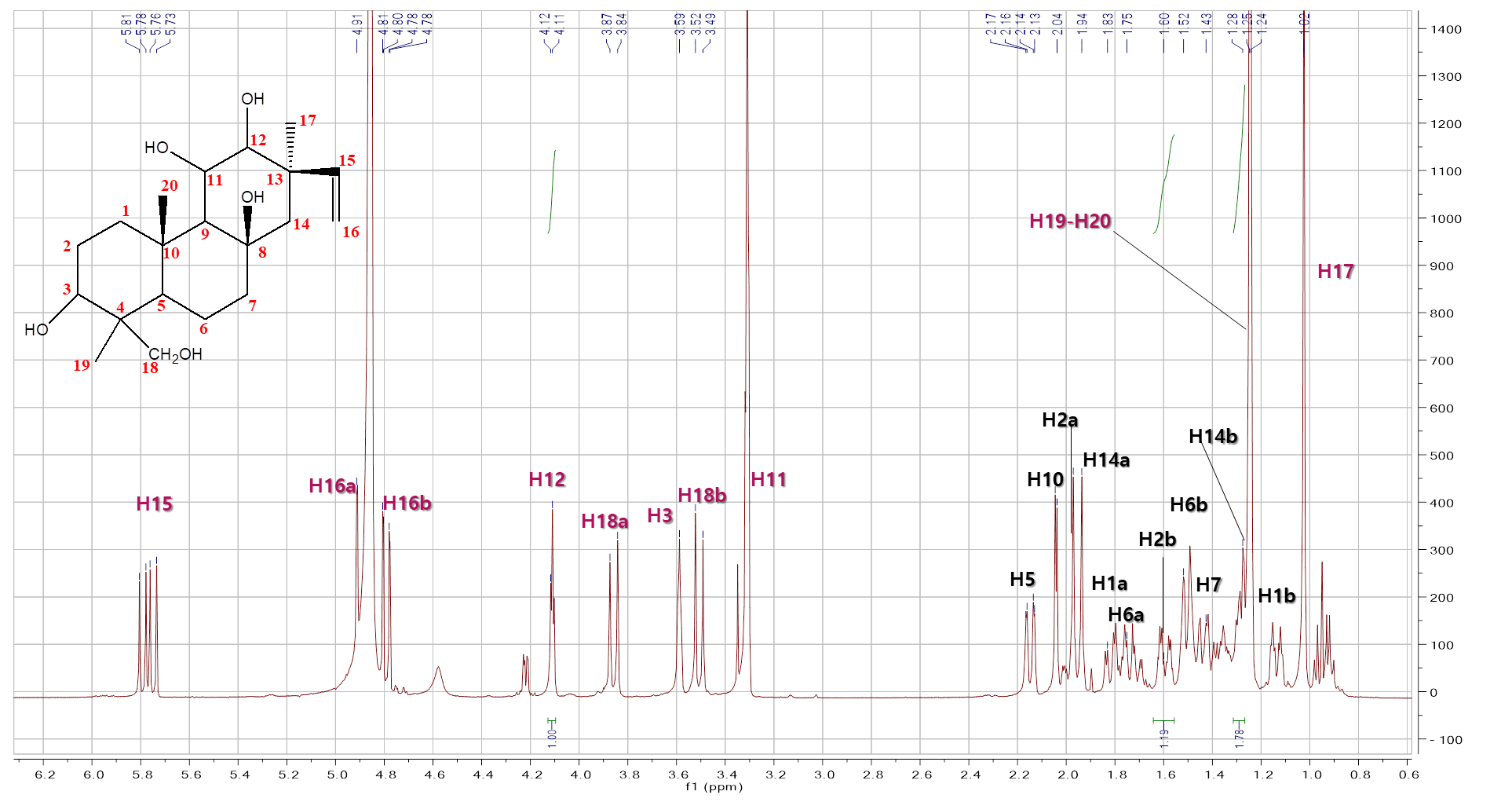


FIGURE S19: ^1^H NMR of Compound **12** (400 MHz, Methanol- *d*_4_)


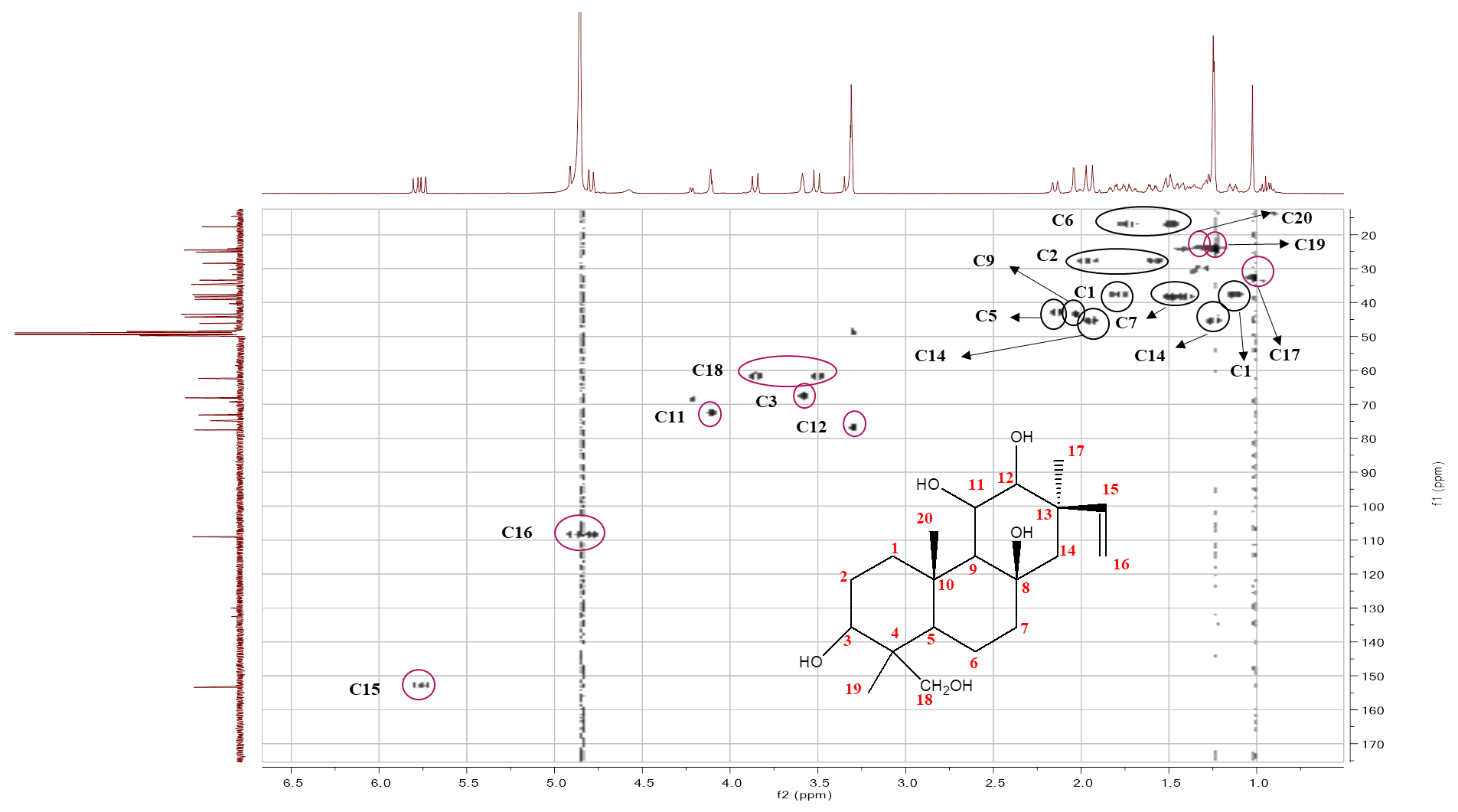
 FIGURE S20: HMQC Correlation of compound 12 (Methanol- *d*_4_)


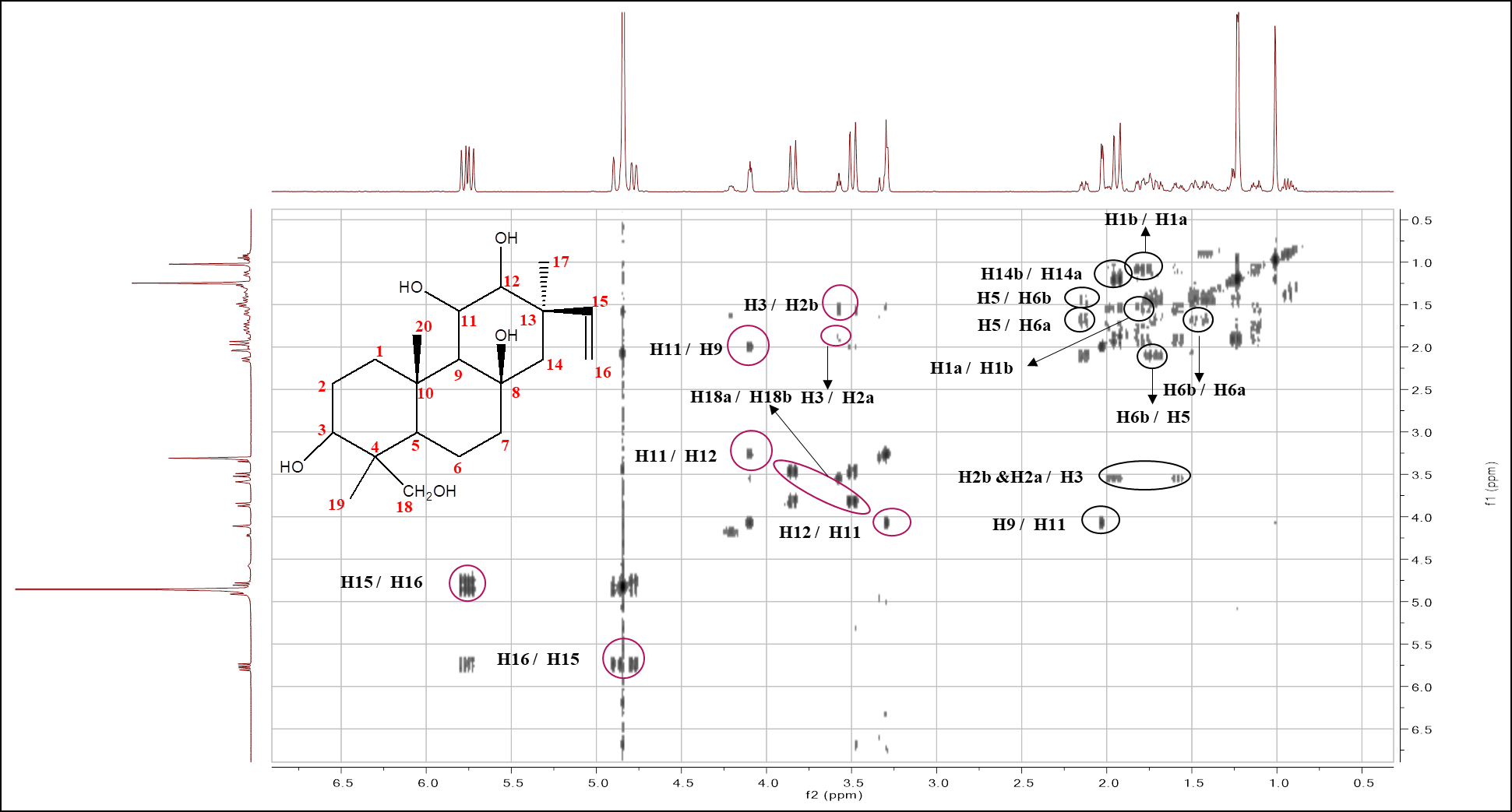


FIGURE S21: COSY Correlation of compound 12 (400 MHz, Methanol- *d*_4_)


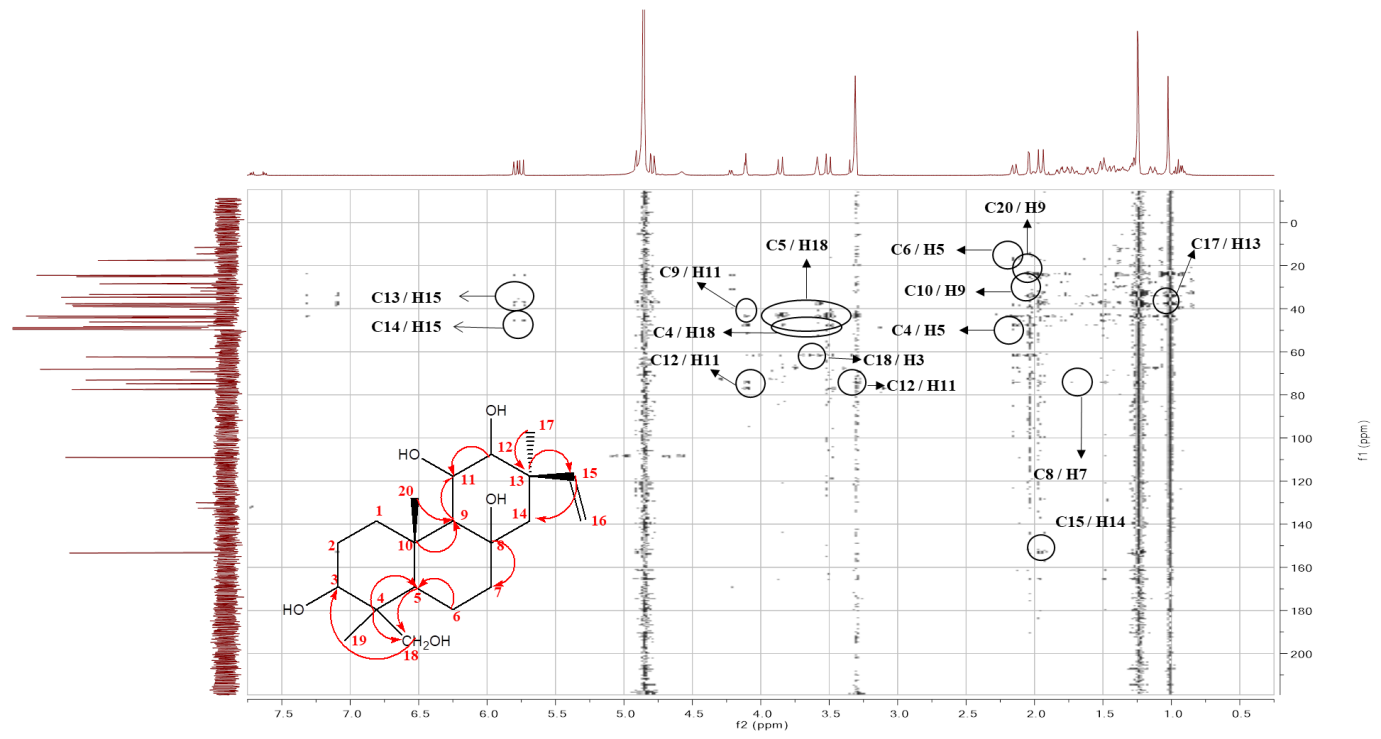


FIGURE S22: HMBC Correlation of compound **12** (Methanol- *d*_4_)


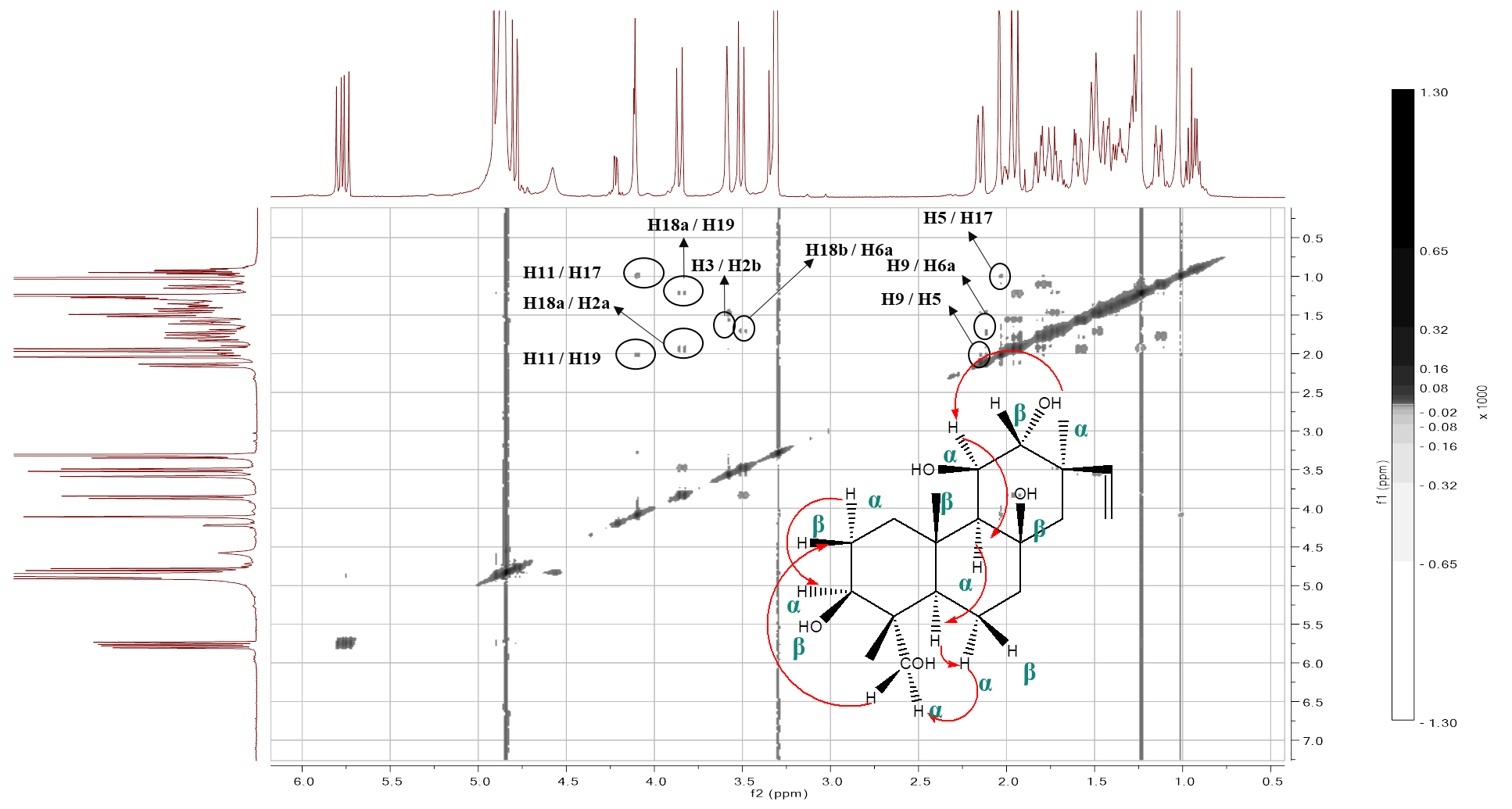


FIGURE S23: ROESY Correlation of compound **12** (Methanol- *d*_4_)


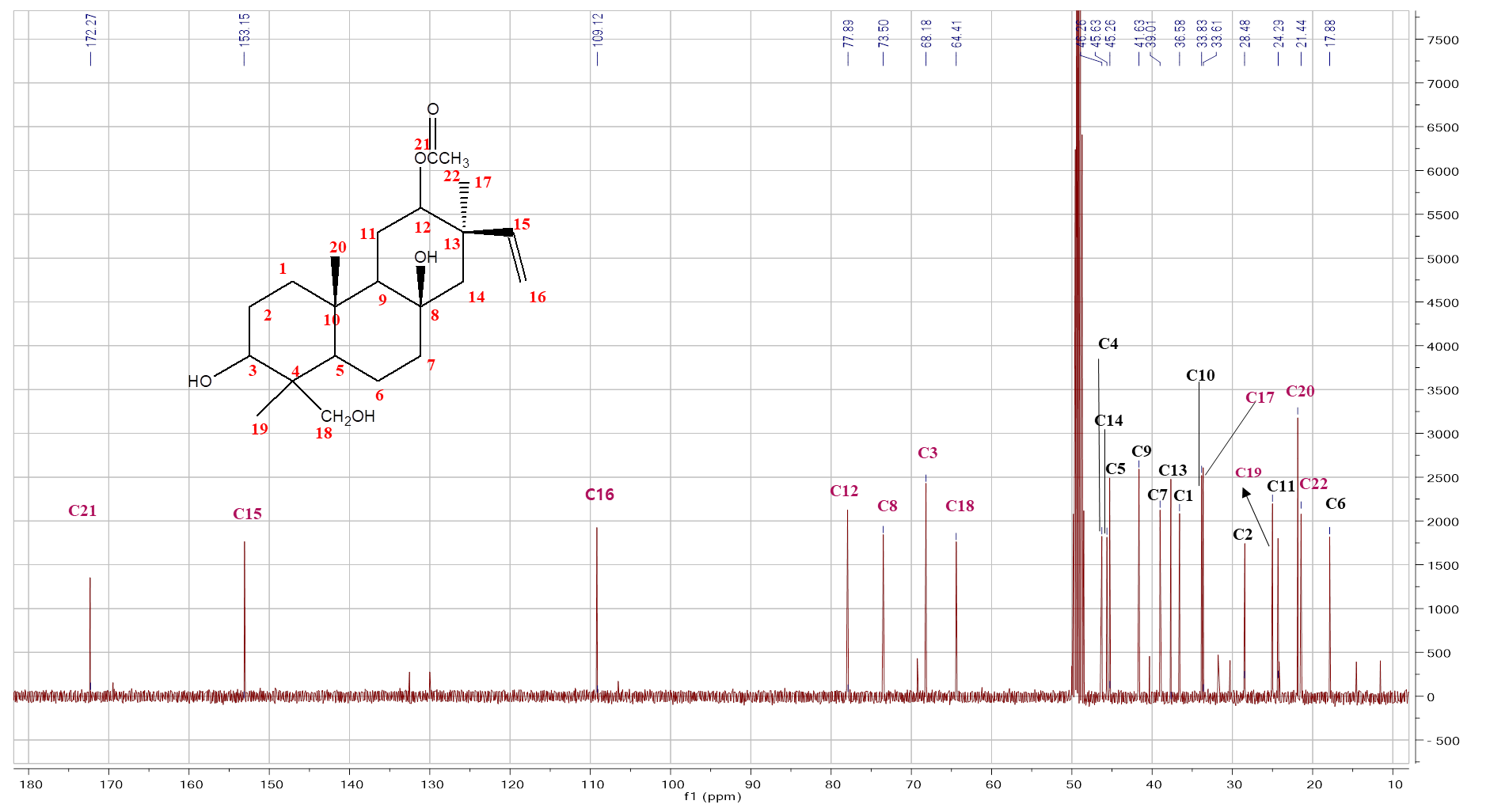


FIGURE S24: ^13^C NMR of Compound **12** (100 MHz, Methanol- *d*_4_)


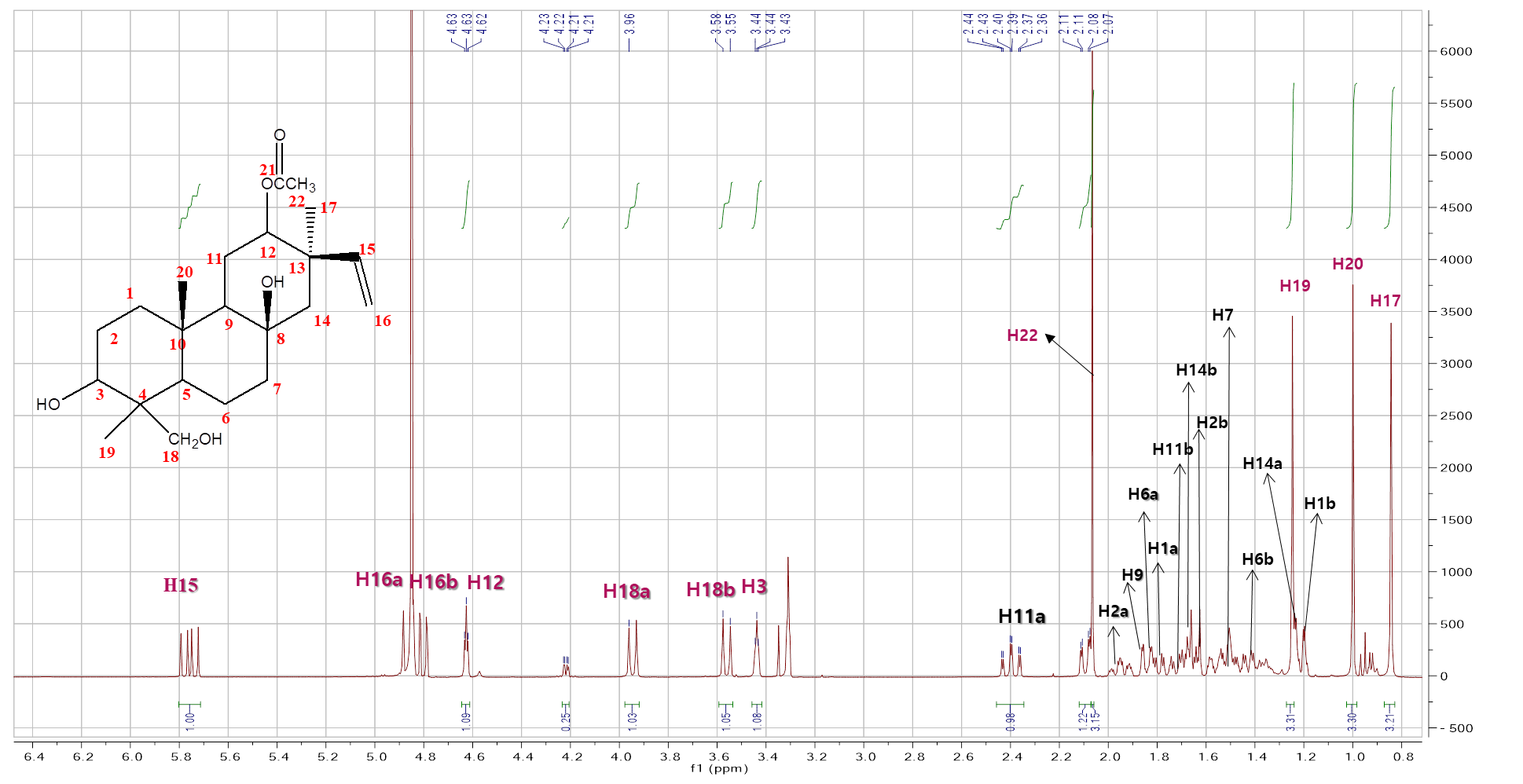


FIGURE S25: ^1^H NMR of Compound **13** (400 MHz, Methanol- *d*_4_)


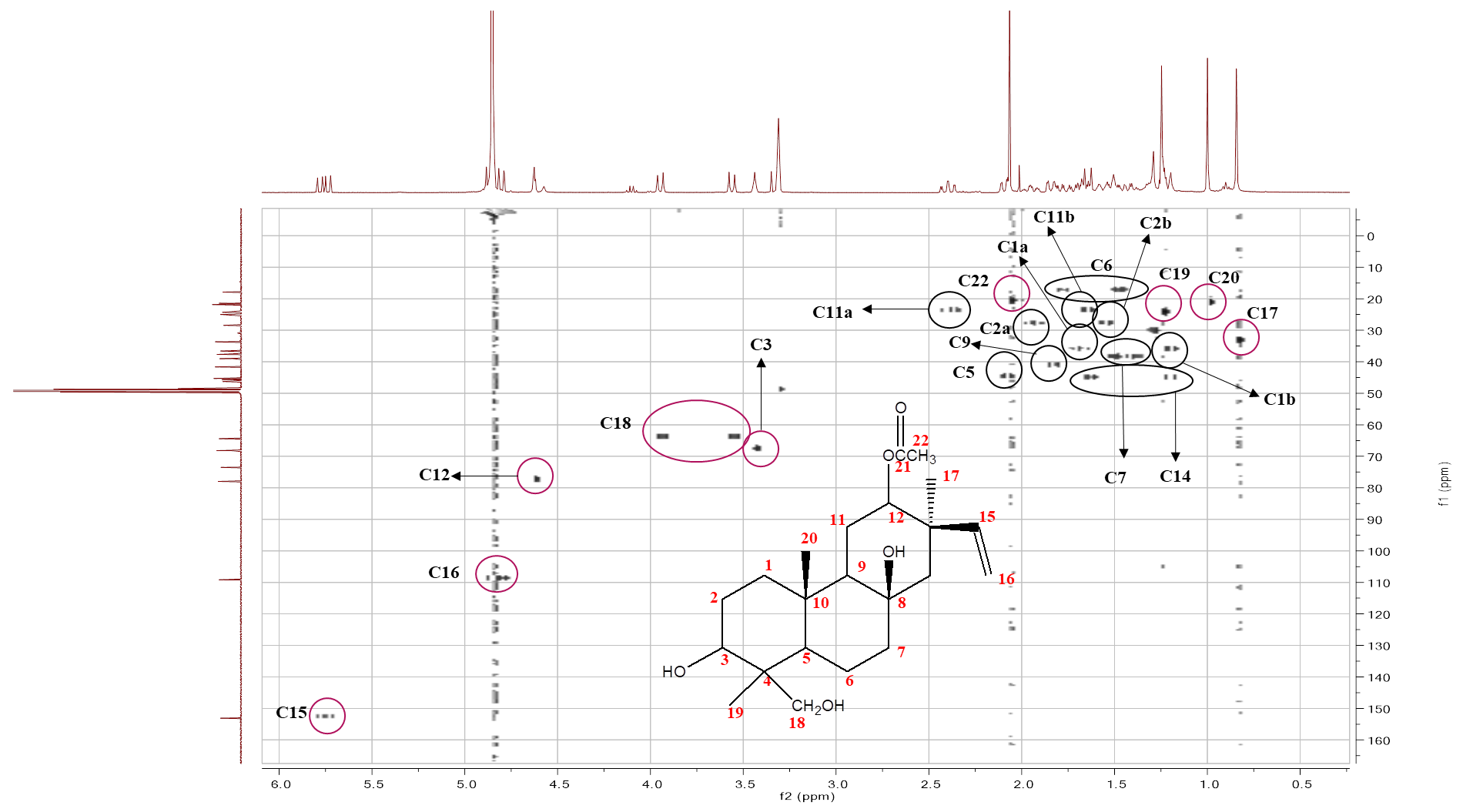


FIGURE S26: HMQC spectrum compound 13 (Methanol- *d*_4_)

**
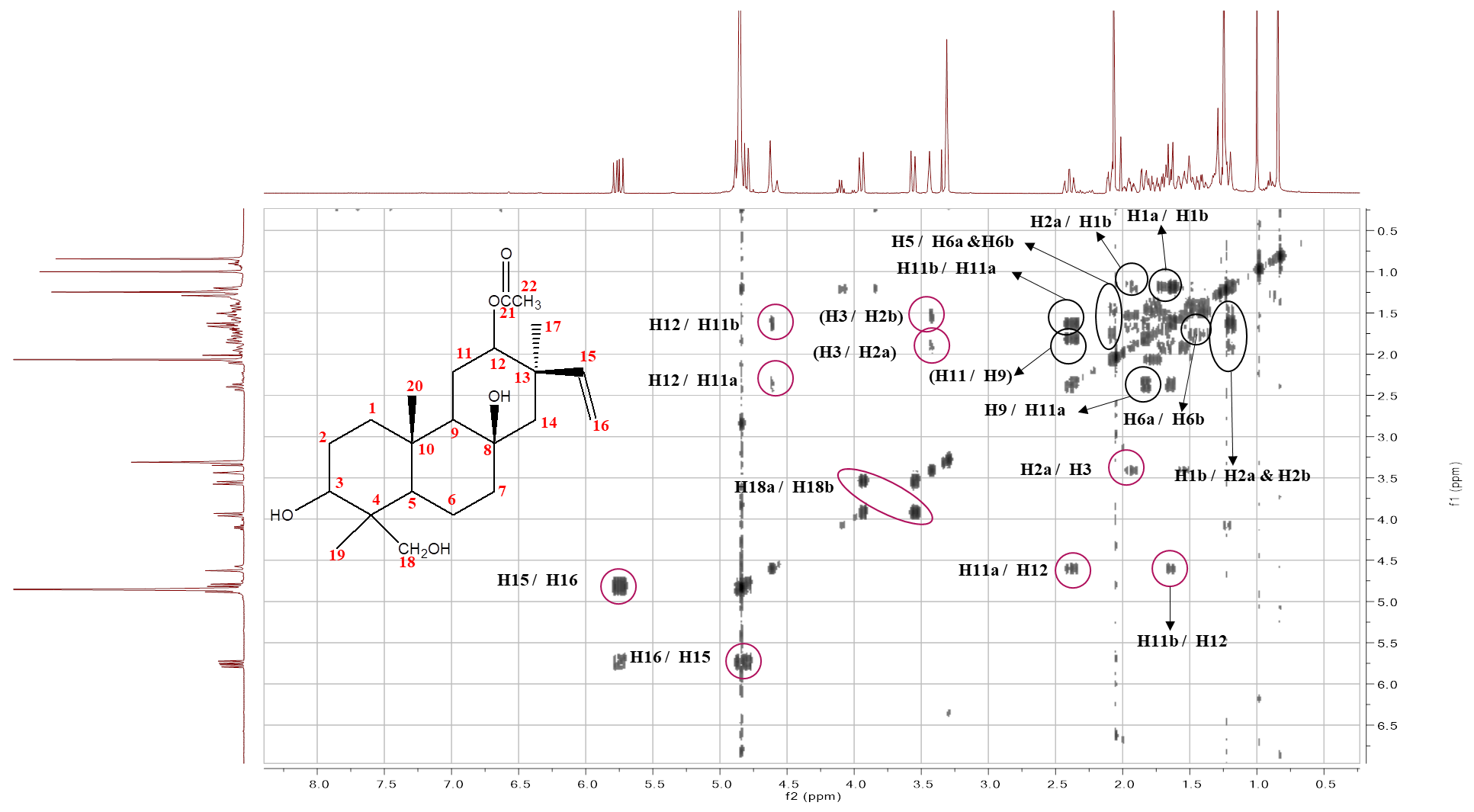
**

FIGURE S27: COSY Correlation of compound **13** (Methanol- *d*_4_)


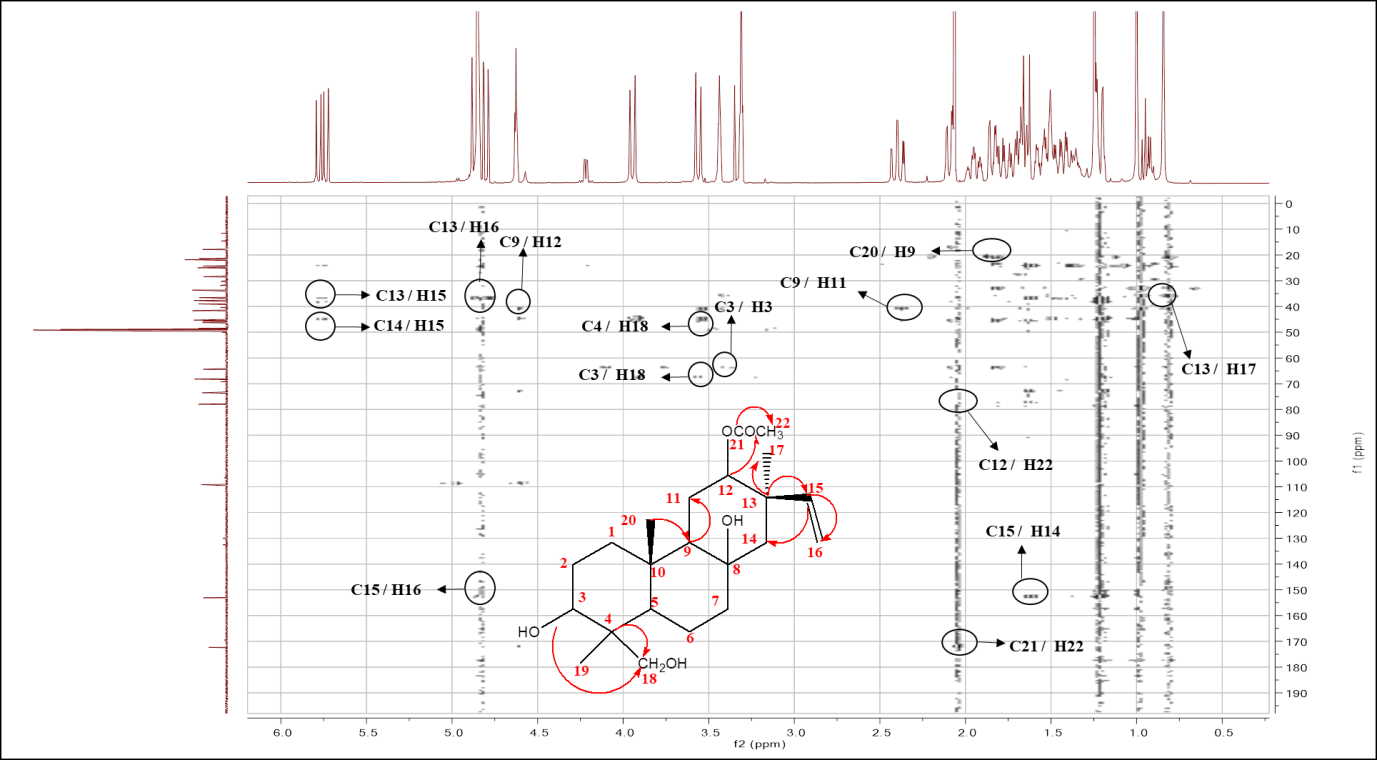


FIGURE S28: HMBC Correlation of compound **13** (Methanol- *d*_4_)

**
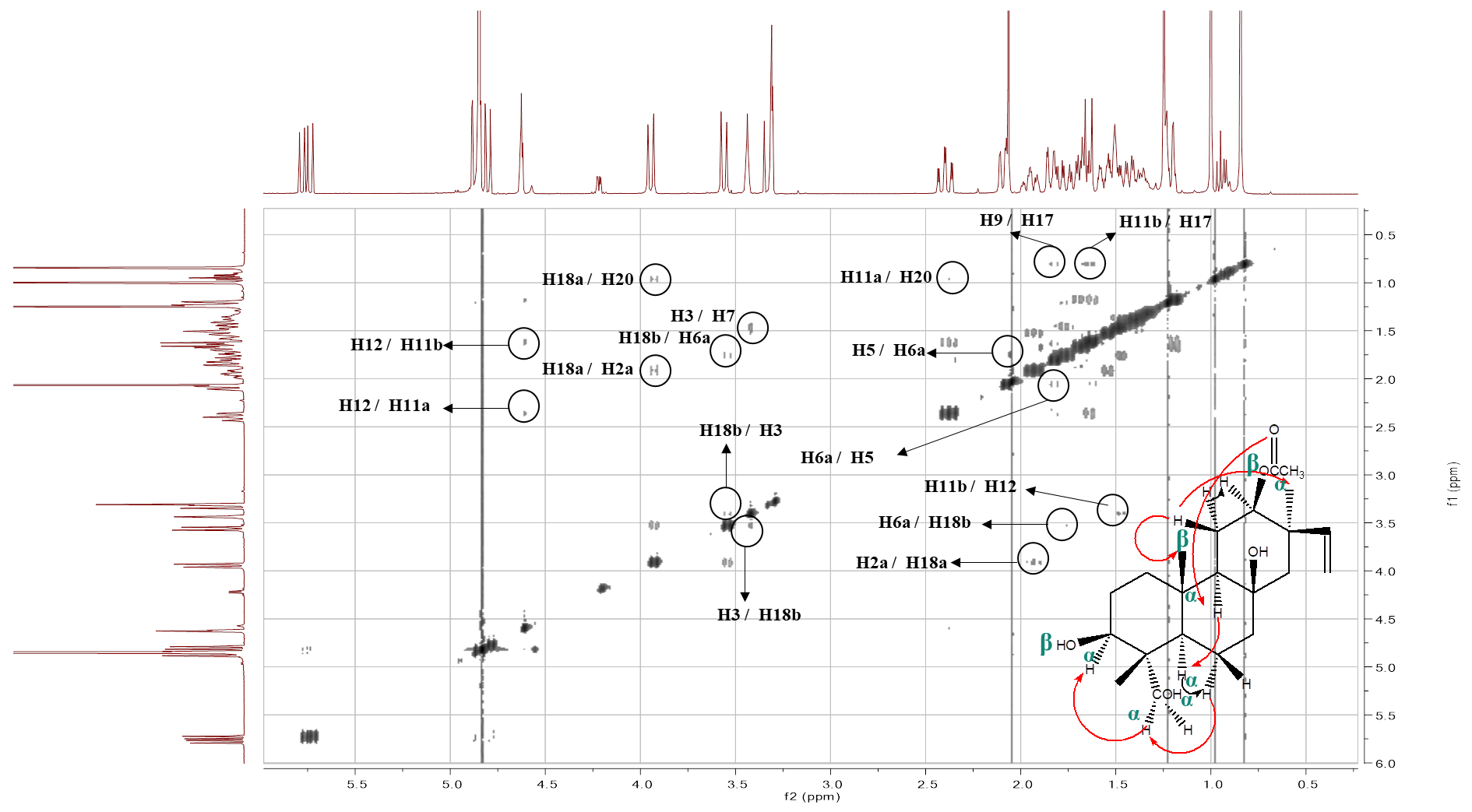
**

FIGURE S29: ROESY Correlation of compound **13** (Methanol- *d*_4_)


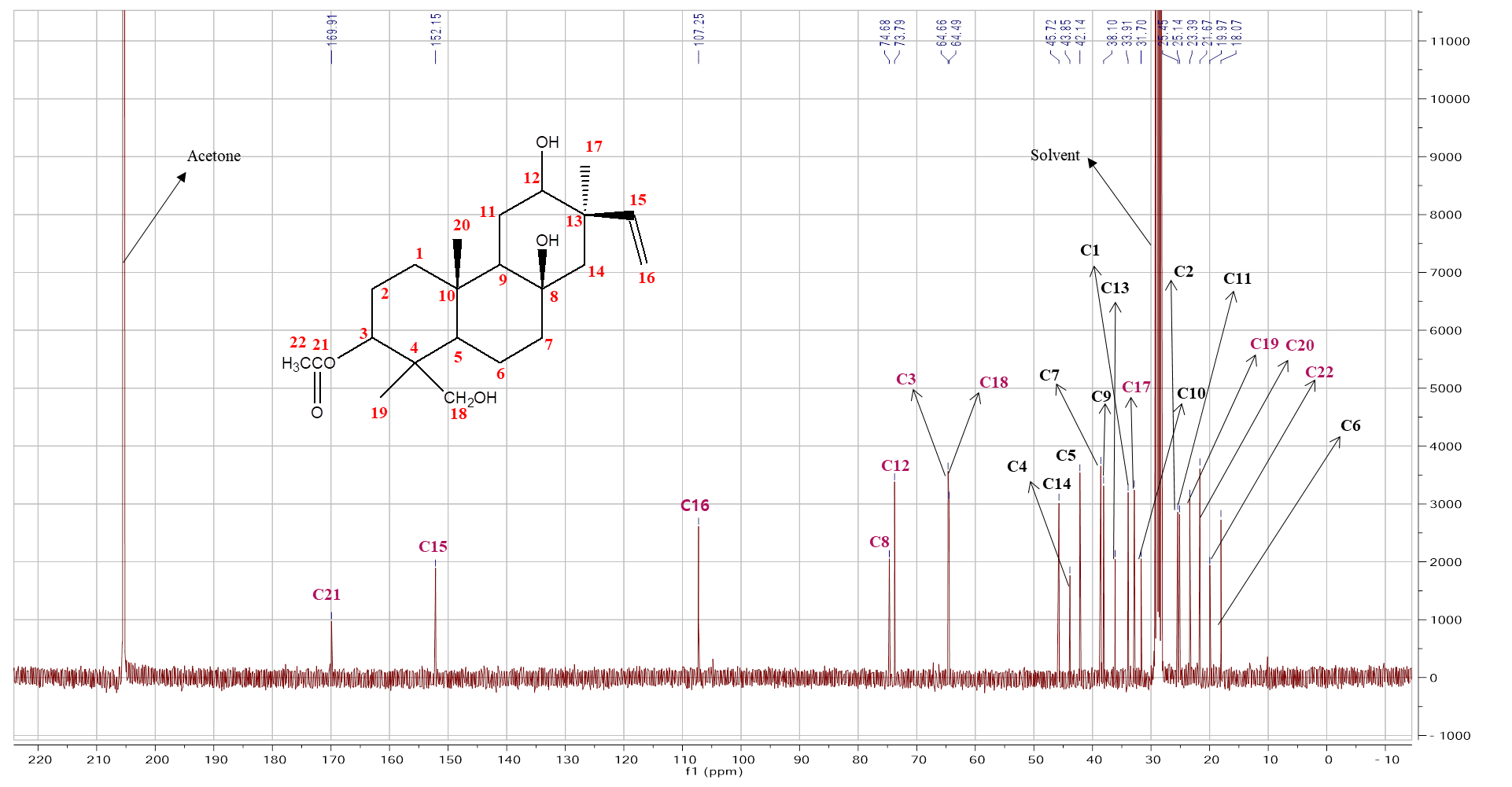


FIGURE S30: ^13^C NMR of Compound **14** (100 MHz, Acetone-*d6*)


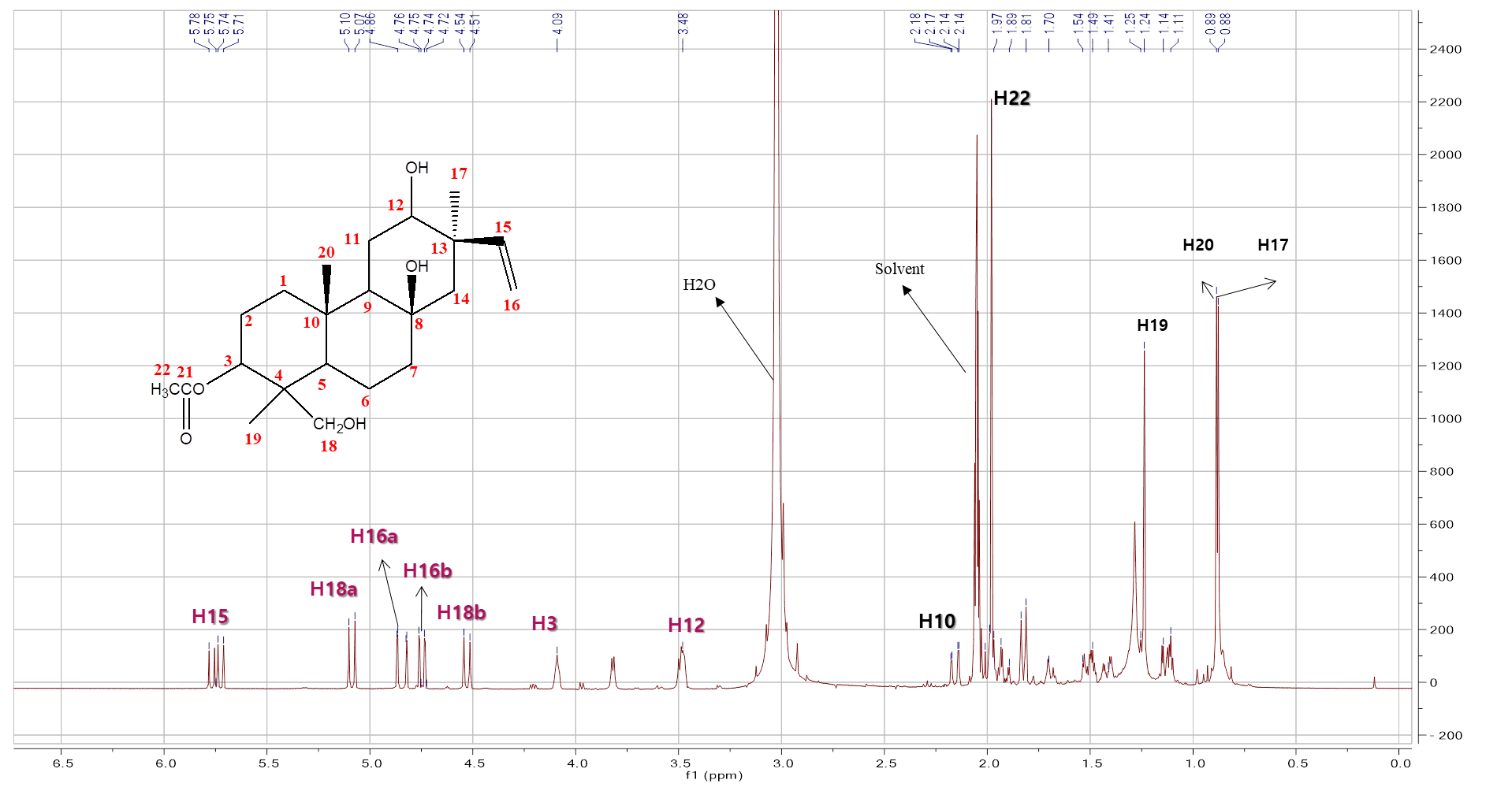


FIGURE S31: ^1^H NMR of Compound **14** (400 MHz, Acetone-*d6*)

**
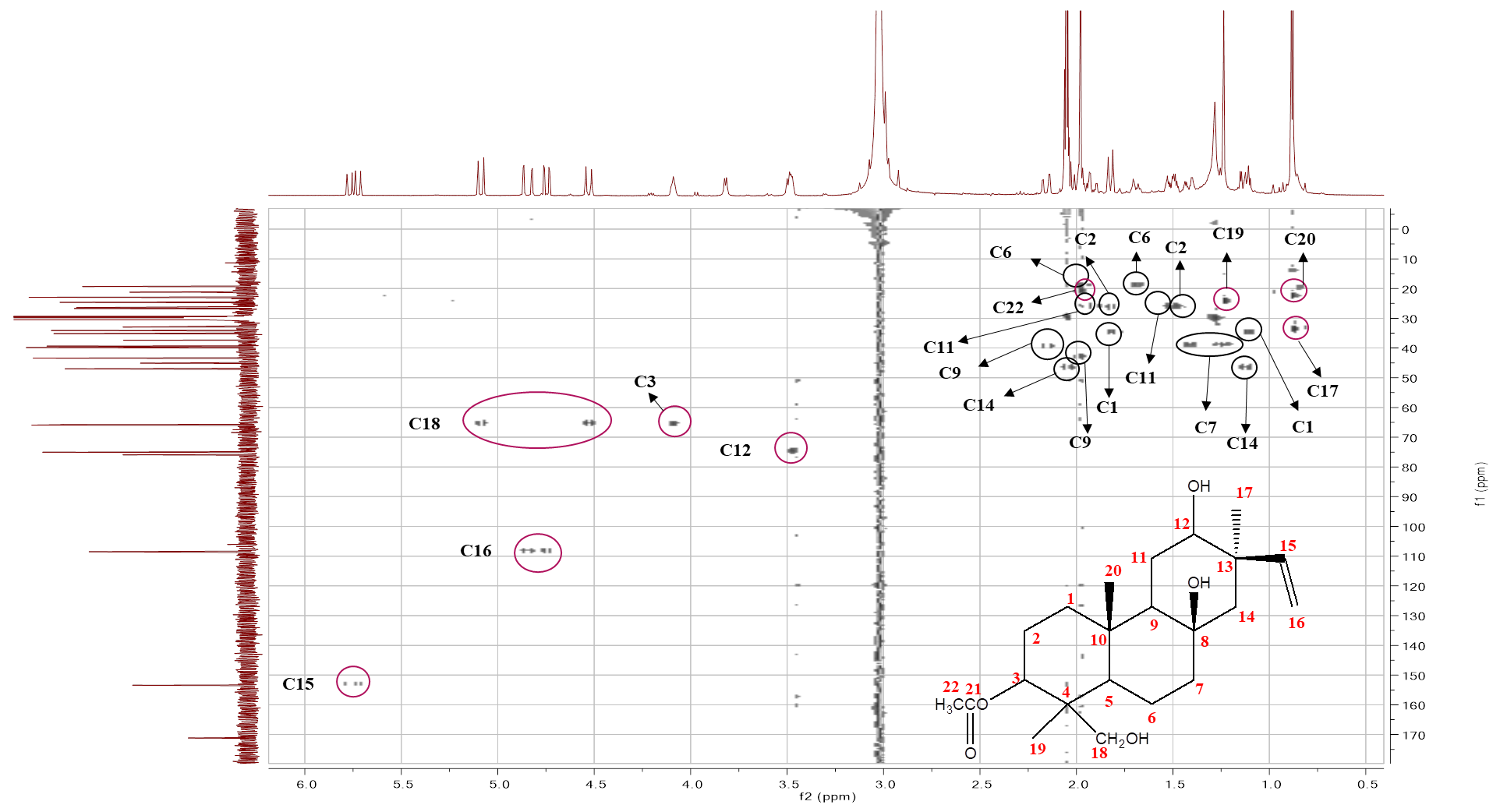
**

FIGURE S32: HMQC Correlation of compound **14** (Acetone- *d*_6_)


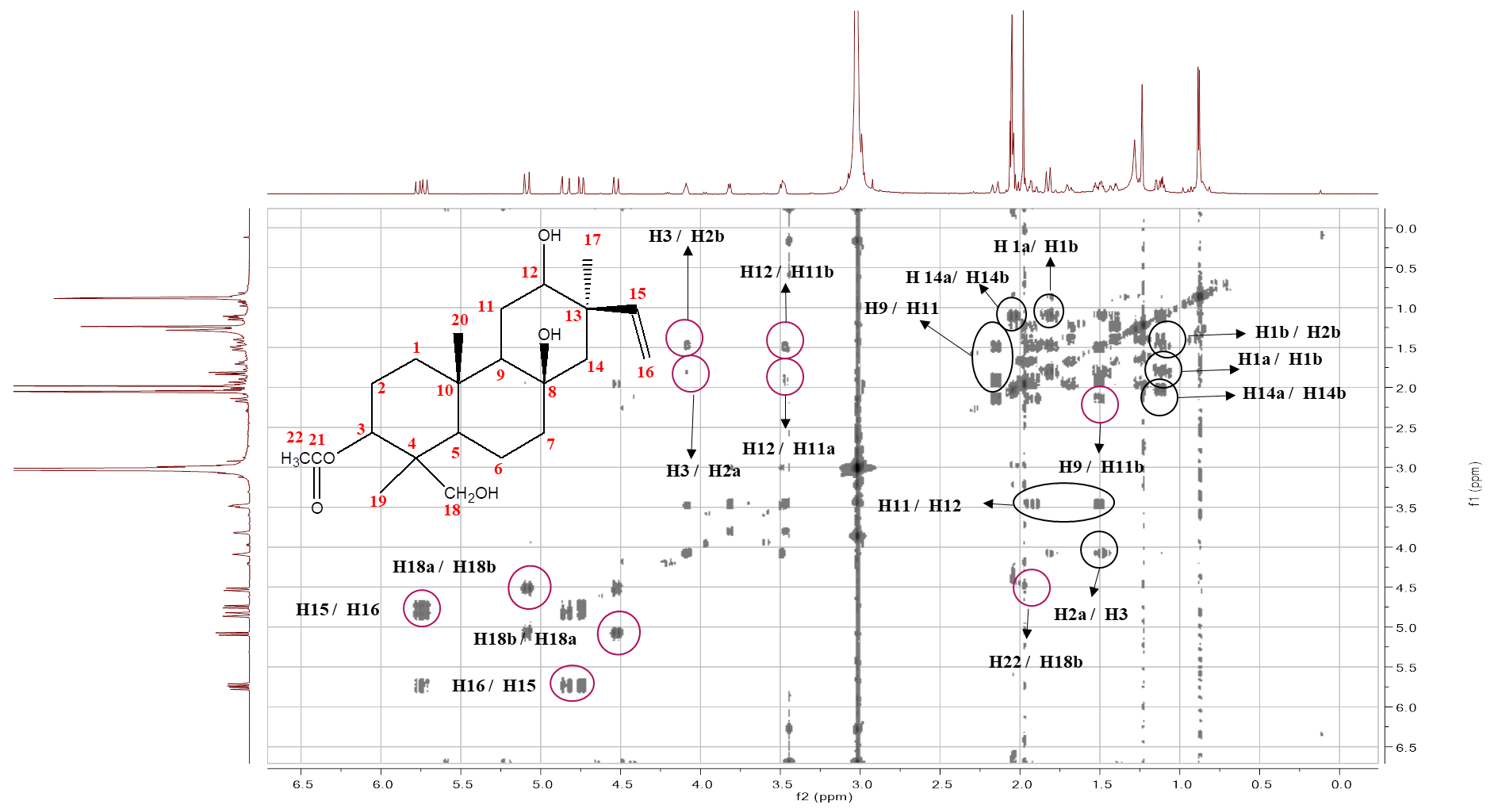


FIGURE S33: COSY Correlation of compound **14** (Acetone- *d*_6_)


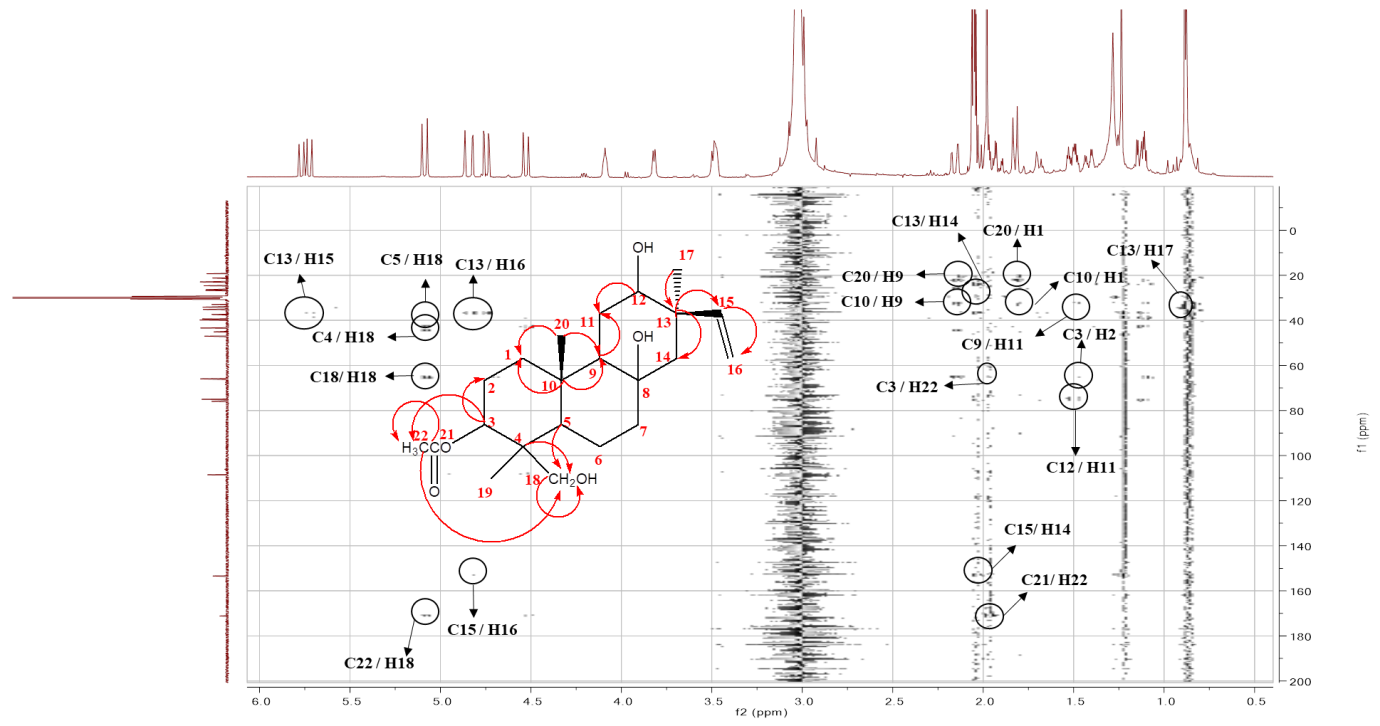


FIGURE S34: HMBC Correlation of compound **14** (Acetone- *d*_6_)


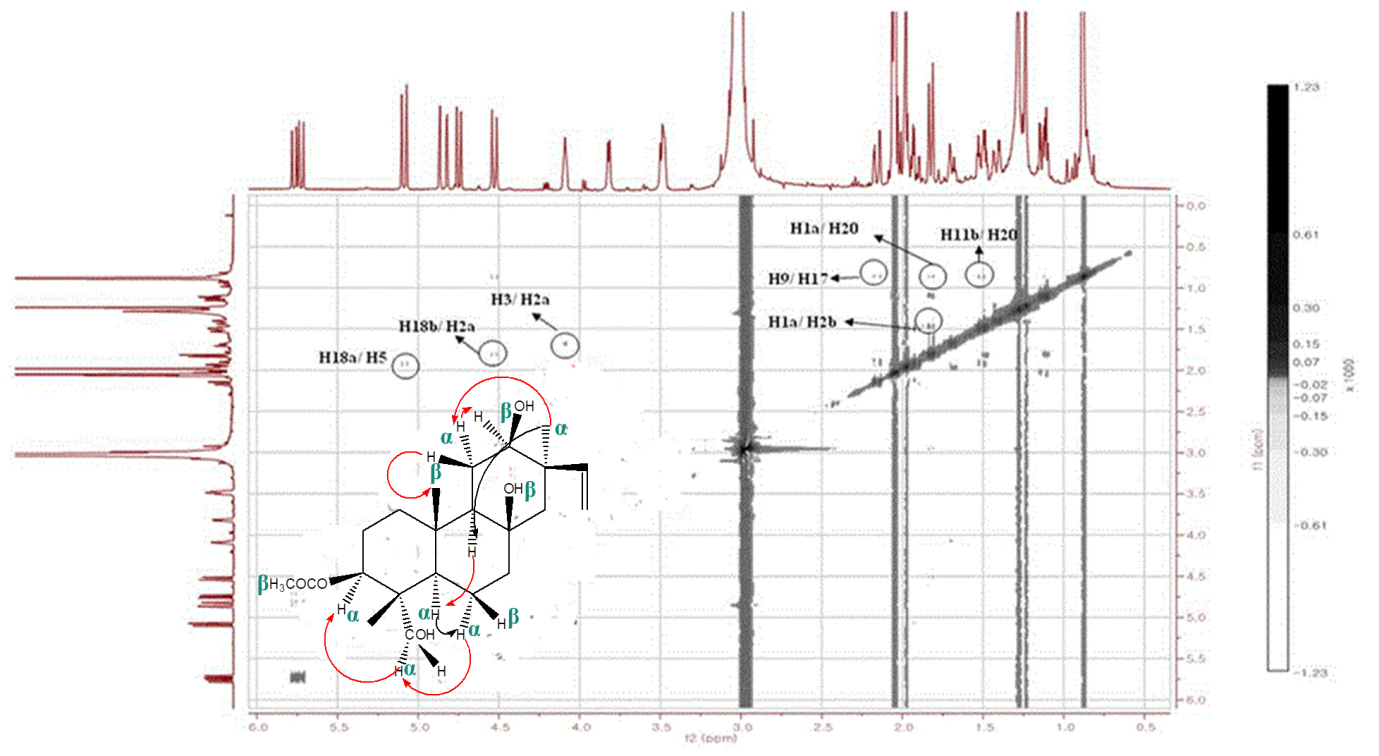


FIGURE S35: ROESY Correlation of compound 14 (Acetone- *d*_6_)

TABLE S1: Identification and microbial analysis of bacterial strains to be examined for antibacterial study of new compounds.

| **SN** | **Microorganisms** | **Gram stain** | **Biochemical Test** | | | | | **Antibiotic Susceptibility** | | |
| --- | --- | --- | --- | --- | --- | --- | --- | --- | --- | --- |
| 1 | *S. aureus (*ATCC 9144) | Positive cocci | **Catalase** | **Oxidase** | **Coagulase** | **Mannitol Fermentation** | | **Sensitive** | **Intermediate** | **Resistant** |
|  |  |  | Positive | Negative | Positive | Positive | | Ciprofloxacin  Gentamycin  Amikacin  Ceftriaxone  Streptomycin | Co-trimaxazole  Chloramphenicol  Cephotaxime | Ampicillin |
| 2 | *S. epidermidis* (ATCC 12228) | Positive cocci | Positive | Negative | Negative | Negative | | Cephotaxime  Chloramphenicol  Gentamycin  Streptomycin sulfate | Amikacin  Chloramphenicol  Ampicillin  Ciprofloxacin | Co-trimaxazole |
| 3 | *P. aeruginosa* (ATCC 27853) | Negative bacilli | **Catalase** | **Oxidase** | **Triple Sugar Iron** | **Indole** | **Citrate** | Amikacin  Gentamycin  Cephotaxime  Vancomycin | Ciprofloxacin  Ceftriaxone | Co-trimaxazole  Ampicillin  Chloramphenicol |
|  |  |  | Positive | Positive | Alkaline/Alkaline | Negative | Positive |  |  |  |
| 4 | *E. oli* (ATCC 14948) | Negative bacilli | Positive | Negative | Acidic/Acidic | Positive | Negative | Chloramphenicol  Co-trimaxazole  Ciprofloxacin  Gentamycin  Cephotaxime  Vancomycin | Amikacin  Ceftriaxone | Ampicillin |
